# Silencing the odorant receptor co-receptor impairs olfactory reception in a sensillum-specific manner in the cockroach

**DOI:** 10.1016/j.isci.2022.104272

**Published:** 2022-04-20

**Authors:** Kosuke Tateishi, Takayuki Watanabe, Hiroshi Nishino, Makoto Mizunami, Hidehiro Watanabe

**Affiliations:** 1 Department of Earth System Science, Fukuoka University, Fukuoka 814-0180, Japan; 2 Research Center for Integrative Evolutionary Science, School of Advanced Sciences, Sokendai-Hayama, Shonan Village, Hayama 240-0193, Japan; 3 Research Institute for Electronic Science, Hokkaido University, Sapporo 060-0812, Japan; 4 Faculty of Science, Hokkaido University, Sapporo 060-0810, Japan

**Keywords:** Entomology, Molecular neuroscience, Sensory neuroscience

## Abstract

Insects detect odors via a large variety of odorant receptors (ORs) expressed in olfactory sensory neurons (OSNs). The insect OR is a heteromeric complex composed of a ligand-specific receptor and the co-receptor (ORco). In this study, we identified the *ORco* gene of the cockroach, *Periplaneta americana* (*PameORco*), and performed RNAi-based functional analysis of *PameORco*. All OSNs in the basiconic sensilla expressed *Pame*ORco and received a large variety of odors including sex pheromones. In trichoid sensilla, a *Pame*ORco-positive OSN was consistently paired with a *Pame*ORco-negative OSN tuned to acids. In adult cockroaches injected with *PameORco* dsRNA at the nymphal stage, the expression of *PameORco*, odor receptions via ORs, and its central processing were strongly suppressed. These results provide new insights into the molecular basis of olfactory reception in the cockroach. The long-lasting and irreversible effects of *PameORco* RNAi would be an effective method for controlling the household pest.

## Highlights

• Whole sequence of *ORco* in the American cockroach (*PameORco*) was characterized
• *Pame*ORco expressed in olfactory sensory neurons in a sensillar type-specific manner
• RNAi chronically and irreversibly suppressed the *PameORco* expression beyond molts
• *Pame*ORco was essential for receptions of sex pheromones and general odors

## Graphical abstract

**Figure:**
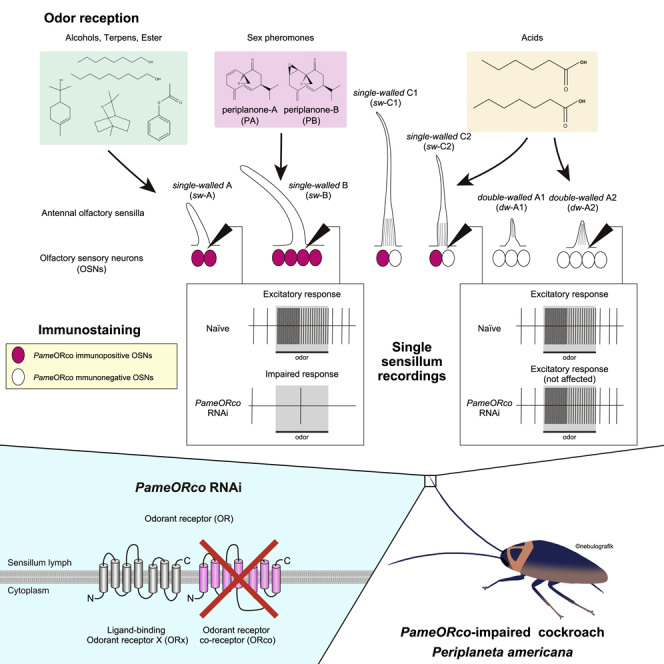

## Introduction

Various species of cockroaches including the American cockroach *Periplaneta americana* are common and widespread pests as vectors of various diseases (Do et al., 2016; Pai et al., 2003). Integrated pest management (IPM), which is a comprehensive strategy for pest control that causes less environmental damage, has received increased attention in recent years. Chemical insecticides and toxic baits have often been used to control cockroaches, but they can also harm non-target organisms. In addition, it is reported in German cockroaches that the excessive use of toxic baits can result in the evolution of pesticide-resistance (Wada-Katsumata et al., 2013). Thus, IPM-based strategies are needed to effectively control the pest cockroaches (Gondhalekar et al., 2021). Because nocturnal cockroaches heavily rely on olfactory cues to search for food and mates, understanding the olfactory system and odor-guided behaviors is essential for optimizing IPM programs for pest cockroaches.

The simple but sophisticated olfactory system in the American cockroach is one of the best models for developing an olfaction-based IPM strategy. To determine how the cockroach encodes vast odors and translates them into odor-guided behaviors, the neural processing of odorants, including pheromones, in *P. americana* has been studied at the cellular levels ranging from peripheral to higher brain centers (Boeckh and Ernst, 1987; Fujimura et al., 1991; Fusca and Kloppenburg, 2021; Nishino et al., 2018; Sass, 1978, 1983; Takahashi et al., 2019; Tichy and Hellwig, 2018; Tichy et al., 2020; Watanabe et al., 2012, 2017). However, the functions of olfactory receptors that confer the response selectivity to a given odor have yet to be identified in the cockroach.

In insects, odorants are generally detected by olfactory sensory neurons (OSNs) in the olfactory sensilla, which are widely distributed in the antennae and maxillary palps (Watanabe et al., 2012; 2018a). Within the olfactory sensilla, odor molecules diffused into the sensillar lymph are received by olfactory receptors expressed on the dendritic cilia of OSNs. The olfactory receptors in insects are classified into two main types belonging to distinct receptor families: odorant receptors (ORs) belonging to the seven-transmembrane type receptor family, and the ionotropic receptors (IRs) belonging to the ionotropic glutamate receptor family (Wicher and Miazzi, 2021). Both ORs and IRs form heteromeric ligand-gated cation channels composed of ligand-specific receptor proteins (ORx or IRx) and their co-receptor proteins (ORco or IRco), respectively. In each insect species, ORx and IRx are highly diverse and responsible for the detection of a set of ligand molecules, whereas co-expressed ORco and IRco are conserved. In ORs, the single ORx/ORco complex is exclusively expressed in a single OSN and confers odor specificity and selectivity of the OSN (Munch and Galizia, 2016).

ORco plays a critical role in the transduction of olfactory signals in OSNs (Carraher et al., 2015). On its own, ORco forms a homotetramer and controls the spontaneous activity of the OSN (Stengl and Funk, 2013; Butterwick et al., 2018). In *Drosophila melanogaster*, phosphorylation and the calmodulin-binding of ORco modulate the olfactory sensitization of the ORx/ORco complex (Guo et al., 2017; Jain et al., 2021; Wicher and Miazzi, 2021). In addition, ORco is essential for transporting and depositing ORx on the dendritic membrane of OSNs (Benton et al., 2006; Stengl and Funk, 2013). Therefore, ORco-impaired insects exhibit anosmia to a given set of odors. In both holometabolous and hemimetabolous insect species including closely related cockroach *Blattella germanica*, the mutagenesis or silencing of *ORco* disrupts odor-guided behaviors, such as orientations to various odorants and pheromone detection (Larsson et al., 2004; DeGennaro et al., 2013; Li et al., 2016; Fandino et al., 2019; Gao et al., 2020; He et al., 2020; Sun et al., 2020). In addition, volatile agonists of ORco elicit behavioral responses in many species of insects (Batra et al., 2019). Therefore, genetic and ligand manipulations of ORco can be useful for controlling insect behaviors in IPM systems (Carey and Carlson, 2011).

In the American cockroach, the response properties of OSNs in each of the olfactory sensilla have been studied for over a half-century (Altner et al., 1977; Sass, 1978, 1983; Boeckh and Ernst, 1987; Fujimura et al., 1991). Olfactory molecules are received by three morphological types of antennal sensilla, the perforated basiconic, trichoid, and grooved basiconic sensilla (Watanabe et al., 2012), which are morphologically analogous to the basiconic, trichoid, and coeloconic sensilla in *D. melanogaster* and other holometabolous insects. The three morphological types of olfactory sensilla are further classified into several subtypes on the basis of the ultrastructural features of the cuticular apparatus (Schaller, 1978). Comprehensive electrophysiological recordings from single olfactory sensilla have revealed that OSNs in different sensillar types differ in their selectivity and sensitivity to ligands (Altner et al., 1977; Boeckh and Ernst, 1987; Fujimura et al., 1991; Sass, 1978, 1983; Tateishi et al., 2020), suggesting that OSNs in different types of olfactory sensilla express different repertories of olfactory receptors. In addition, in the cockroach, two parallel olfactory pathways stem from OSNs in perforated basiconic and trichoid/grooved basiconic sensilla, which feed information to partly overlapping but distinct types of Kenyon cells in the mushroom body via distinct types of projection neurons (Watanabe et al., 2010a, 2012, 2017; Takahashi et al., 2019). Therefore, general odors are processed from peripheral to higher brain centers in a sensillum-specific manner.

Sex pheromones emitted by adult females strongly attract adult males and elicit sexual behaviors in the American cockroach (Seelinger and Gagel, 1985). Therefore, the peripheral reception and central processing of sex pheromones have been extensively studied in the cockroach. Two major components of sex pheromones in the cockroach, periplanone-A (PA) and -B (PB), are specifically received by PA-sensory neuron (PA-SN) and PB-SN in the longer perforated basiconic sensilla, termed *single-walled* B (*sw*-B) sensilla (Persoons et al., 1990; Sass, 1983). PA-SN and PB-SN send axons to two macroglomeruli, A- and B-glomerulus, in the adult male antennal lobe (Watanabe et al., 2012; 2018a), and synapse onto PA- and PB-responsive projection neurons (PNs), respectively (Nishino et al., 2018). These PNs terminate in specific regions of the ipsilateral mushroom body and the lateral horn (Nishino et al., 2018). Thus, sex pheromone processing from peripheral to higher brain centers is well understood in the cockroach, but the transduction mechanisms of sex pheromones have yet to be characterized.

Recently, a 3.38-Gb draft genome sequence of *P. americana* was published, and 154 candidate ORs and 640 candidate IRs were annotated (Li et al., 2018). Among them, a transcriptome analysis revealed that 96 ORs and 53 IRs are expressed in cockroach antennae (Chen et al., 2016). However, detailed information on the gene sequences of each of the olfactory receptors was lacking in previous studies, precluding the functional analysis. In this study, we identified the *ORco* gene in *P. americana* (*Pam*e*ORco*), generated anti-*Pame*ORco antiserum for immunostaining, and performed systemic RNAi-based functional analysis of *PameORco* for the cockroach olfaction. The antiserum of *Pame*ORco selectively labeled OSNs in a sensillum-specific manner, including those in sex pheromone-responsive *sw*-B sensilla. Impairment of *PameORco* expression resulted in a significant decrease in the olfactory responses of *Pame*ORco-immunopositive OSNs. In particular, the effect of *PameORco* RNAi impaired sex pheromone responses by affecting peripheral PB-SNs, central PB-responsive PNs where a large number of PB-SNs converge, and sexual behaviors to PB. This is the first study to reveal the fundamental molecular process of odor transduction in the olfaction of the American cockroach, the animal model for IPM.

## Results

### Identification of *PameORco*

**Figure 1:**
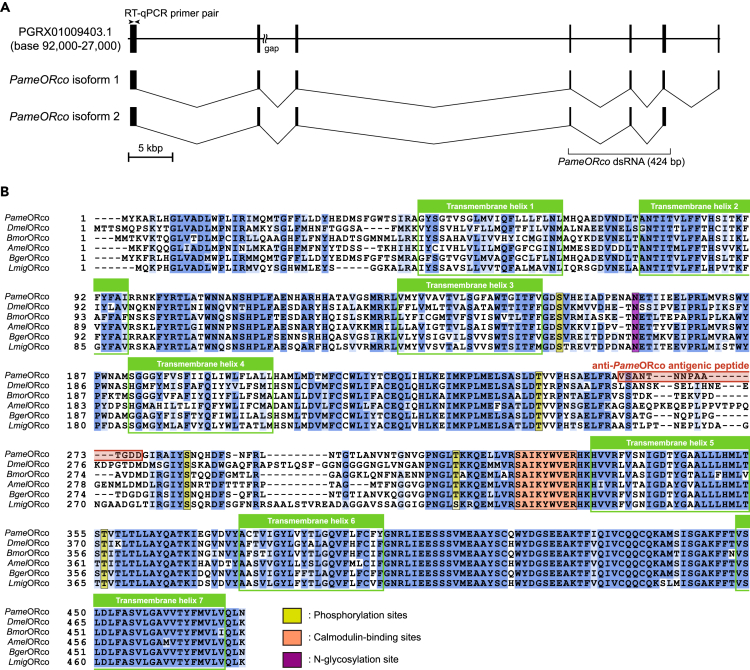
Identification of *Periplaneta americana ORco* (*PameORco*) (A) Organization of *PameORco*. The positions of primers used for RT-qPCR analysis, and the target region of dsRNA for *PameORco* RNAi are illustrated. (B) Amino acid sequence alignment of *Pame*ORco isoform 2 and its homologs in five different insect species. The letters are shaded by alignment strength. The positions of seven transmembrane helixes, putative phosphorylation sites (yellow shaded), putative calmodulin-binding site (orange shaded), and putative N-linked glycosylation site (purple shaded) are illustrated. The polyclonal anti-*Pame*ORco antiserum was generated against the 14-a.a. synthetic antigenic peptide located within the second intracellular loop (red shaded). GenBank IDs: *PameORco* isoform 1, LC657818; and 2, LC657819; *Drosophila melanogaster* ORco (*Dmel*ORco), Q9VNB5; *Bombyx mori* ORco (*Bmor*ORco), NP_001,037,060; *Apis mellifera* ORco (*Amel*ORco), NP_001,128,415; *Blattella germanica* ORco (*Bger*ORco), PSN39983; and *Locusta migratoria* ORco (*Lmig*ORco), ALD51504. See also Figure S1.

A tbalstn search revealed that a single *ORco* gene is encoded by the genome of *P. americana*. The coding region of the *ORco* gene is encoded by seven exons spanning ∼6.5 kb in the genome (GenBank ID: base 91,777-33,081 of PGRX01009403.1; Figure 1A). Next, we amplified 1444-bp and 1495-bp cDNA fragments encoding two *PameORco* isoforms (471 a.a. and 472 a.a., respectively) in the cockroach antennae using reverse transcription PCR (RT-PCR)-based cDNA cloning (GenBank ID: LC657818 and LC657819). The *PameORco* isoforms contain seven transmembrane helixes and putative phosphorylation sites (Guo et al., 2017), a putative calmodulin-binding site (Jain et al., 2021), and a putative N-linked glycosylation site (Lundin et al., 2007) conserved in insect ORco homologs (Figure 1B). The two *Pame*ORco isoforms share the N-terminal 456 a.a. and differ at their C-terminal end corresponding to the C-terminal half of the seventh transmembrane helix. The C-terminal tail of *PameORco* isoform 1 contains multiple hydrophilic residues, which is not predicted as transmembrane region by the TMHMM2.0 algorithm. With these data, we concluded that the *PameORco* isoform 1 is a missplicing product of the *PameORco* gene, and does not encode functional *PameORco* protein. Therefore, we focused on the *PameORco* isoform 2 in this study. A molecular phylogenetic analysis of insect ORco homologs confirmed that *Pame*ORco is closely related to the ORco homologs of other hemimetabolous insect species (Figure S1).

### Anti-*Pame*ORco antiserum selectively labeled OSNs in specific olfactory sensilla

**Figure 2:**
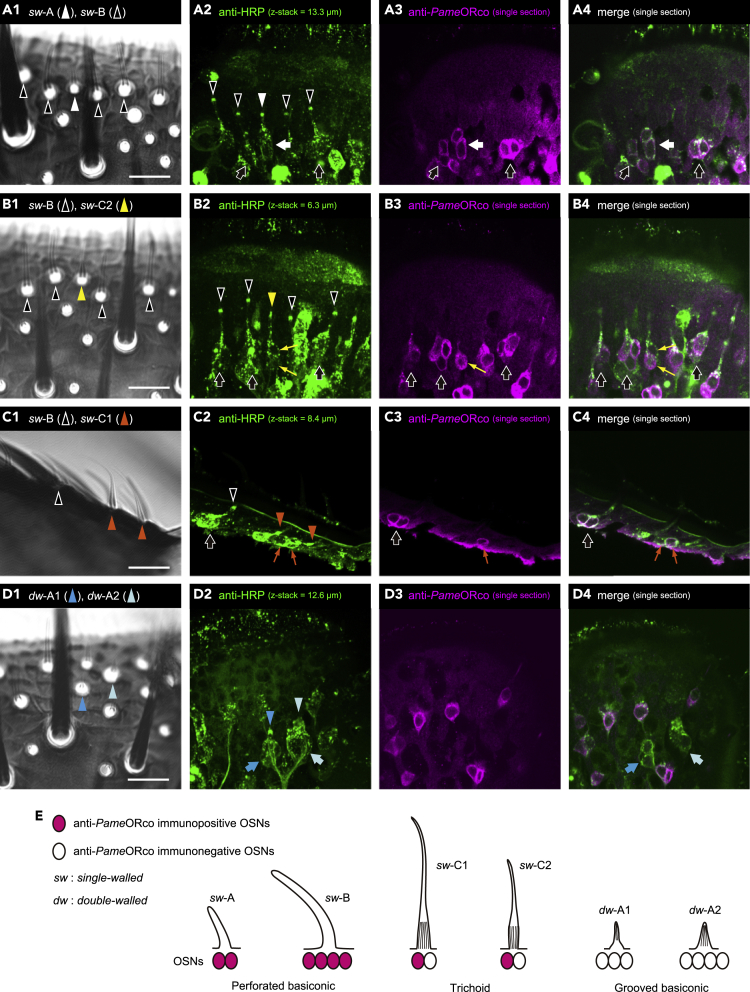
Sensillar type-specific distribution patterns of *Pame*ORco-expressing OSNs (A–D) Anti-*Pame*ORco-immunopositive and immunonegative OSNs in all morphological types of antennal olfactory sensilla. Six morphological types of antennal olfactory sensilla are unambiguously identified via differential interference contrast (DIC) observations and indicated by different colored arrowheads (A1–D1). All antennal sensory neurons labeled by anti-HRP antibody are green-colored (A2–D2 and A4–B4), whereas anti-*Pame*ORco-immunopositive OSNs are magenta-colored (A3–D3 and A4–B4). Sensilla and corresponding OSNs are denoted as arrowheads and arrows of the same color, respectively. Two OSNs in single trichoid sensilla (*sw*-C1 and *sw*-C2 sensilla) are indicated by thin arrows (B2–B4 and C2–C4). In single trichoid sensilla, a *Pame*ORco-positive OSN was consistently paired with a *Pame*ORco-negative OSN (thin arrows in B3,4 and C3,4). Scale bar = 20 μm. See also Figure S2 and Videos S1, S2, S3, and S4. (E) A schematic overview of sensillar type-specific distribution patterns of *Pame*ORco-expressing OSNs.

To examine the distribution of *Pame*ORco-expressing OSNs in the cockroach antennae, we generated anti-*Pame*ORco antiserum in a guinea pig and conducted immunohistochemistry. The antennae of adult male cockroaches were double-immunolabeled with the anti-*Pame*ORco antiserum and the polyclonal anti-HRP antibody generated in a rabbit. The anti-HRP antibody selectively labels sensory neurons in cockroach antennae (Watanabe et al., 2018a). The types of olfactory sensilla were unambiguously identified by differential interference contrast (DIC) optics (Figures 2A1–2D1, Videos S1–S4). We investigated the distributions of *Pame*ORco-expressing OSNs in three types of olfactory sensilla; perforated basiconic, trichoid (Figures 2A, 2B, and 2C), and grooved basiconic sensilla (Figure 2D). Perforated basiconic sensilla are further categorized into two subtypes on the basis of their lengths; shorter *single-walled* A (*sw*-A) sensilla and sex pheromone-responsive longer *sw*-B sensilla (Schaller, 1978; Watanabe et al., 2018a). Anti-HRP antibody revealed two and four OSNs in single *sw*-A and *sw*-B sensilla, respectively, and these OSNs were also labeled by anti-*Pame*ORco antiserum (Figure 2A). However, OSNs in the grooved basiconic sensilla were not labeled by anti-*Pame*ORco antiserum regardless of the subtypes (three OSNs in *double-walled* A1 (*dw*-A1) and four OSNs in *dw*-A2 sensilla in Figure 2D). The longer and shorter trichoid sensilla were termed *sw*-C1 and *sw*-C2 sensilla, respectively (Watanabe et al., 2012). Anti-HRP antibody revealed that single trichoid sensilla had two OSNs. Regardless of subtype, a *Pame*ORco-positive OSN was consistently paired with a *Pame*ORco-negative OSN in single trichoid sensilla (Figures 2B and 2C). Mechanosensory and contact chemosensory neurons in the chaetic sensilla, and hygrosensory and thermosensory neurons in the capitular sensilla were not labeled by anti-*Pame*ORco antiserum (Figure S2). The distribution patterns of anti-*Pame*ORco immunoreactive OSNs are summarized in schemes (Figure 2E).

### *PameORco* RNAi and its effectiveness

**Figure 3:**
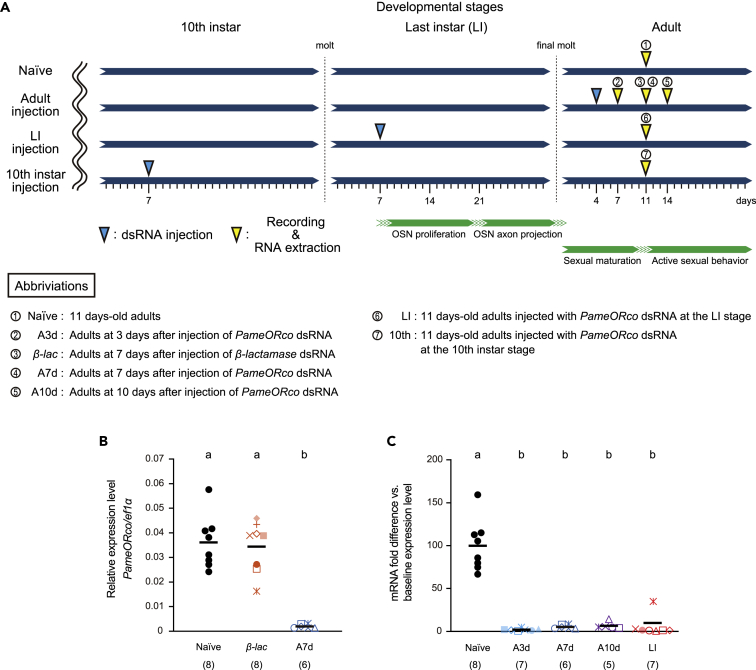
Experimental conditions for *PameORco* RNAi and *PameORco* expressions (A) Experimental conditions for *PameORco* RNAi. Experimental conditions are summarized in the scheme and abbreviations. At arbitrary selected days after dsRNA injection, RNAi effects were examined by single sensillum recordings and RT-qPCRs (yellow arrowheads). Abbreviations denoted in the scheme are used in the following figures. Developmental processes of OSNs and sexual maturation are indicated by green bars (Silverman, 1977; Watanabe et al., 2018a). (B) *PameORco* expressions in Naive, *β-lac*, and *PameORco* dsRNA-injected cockroaches. In dsRNA-injected cockroaches, RT-qPCRs were performed at 7 days after dsRNA injection. The expression levels of *PameORco* mRNA were normalized with that of *PameEf1α* mRNA. (C) *PameORco* expressions across the different post-injection periods of dsRNA. The expression levels of *PameORco* were normalized to the average of those of Naive cockroaches (baseline expression level). In (B and C), the sample number is noted in parentheses and black bars indicate means. The different letters above each plot indicate significant differences (ANOVA post-hoc Tukey-Kramer test; p < 0.05). Samples from eight Naive cockroaches (black dots in B and C) were specifically prepared for the RT-qPCR analysis, but those obtained from RNAi cockroaches were also used in the following electrophysiological experiments. Therefore, throughout following figures, results obtained from the same individuals are represented as symbols of the same color and shape.

To validate the effectiveness of *PameORco* RNAi, we conducted RT-qPCR analysis to examine the expression level of *PameORco* in the antennae at different time points after dsRNA injection (Figure 3). Nymphal and adult male cockroaches were injected with 4 μg of dsRNA of *PameORco* or *Escherichia coli β-lactamase* (*β-lac*; negative control), and the expression level of *PameORco* in both antennae was quantified at arbitrarily selected days after dsRNA injection (Figure 3A). In the RT-qPCR, the expression levels of *PameORco* were normalized with that of *elongation factor 1-alpha* in *P. americana* (*PameEf1α*; GenBank ID: LC657820). There was a significant reduction of the expression levels of *PameORco* in *PameORco* dsRNA-injected cockroaches compared to Naive or *β-lac* dsRNA-injected cockroaches (Figures 3B and 3C). Because expression levels of *PameORco* were not different between *β-lac* dsRNA-injected and Naive cockroaches (Figure 3B), the injection procedure did not affect the expression of *PameORco*. Compared to the Naive cockroaches, the expression levels of *PameORco* were reduced to 2.1 ± 1.5% at 3 days after *PameORco* dsRNA injection and the strong suppression of *PameORco* expression was observed across different post-dsRNA injection periods (Figure 3C). The effects of *PameORco* RNAi were maintained beyond developmental stages; adult cockroaches injected with *PameORco* dsRNA at the last instar stage also exhibited the prominent RNAi effect (Figure 3C).

### Silencing of the *PameORco* expression impaired sex pheromone reception

**Figure 4:**
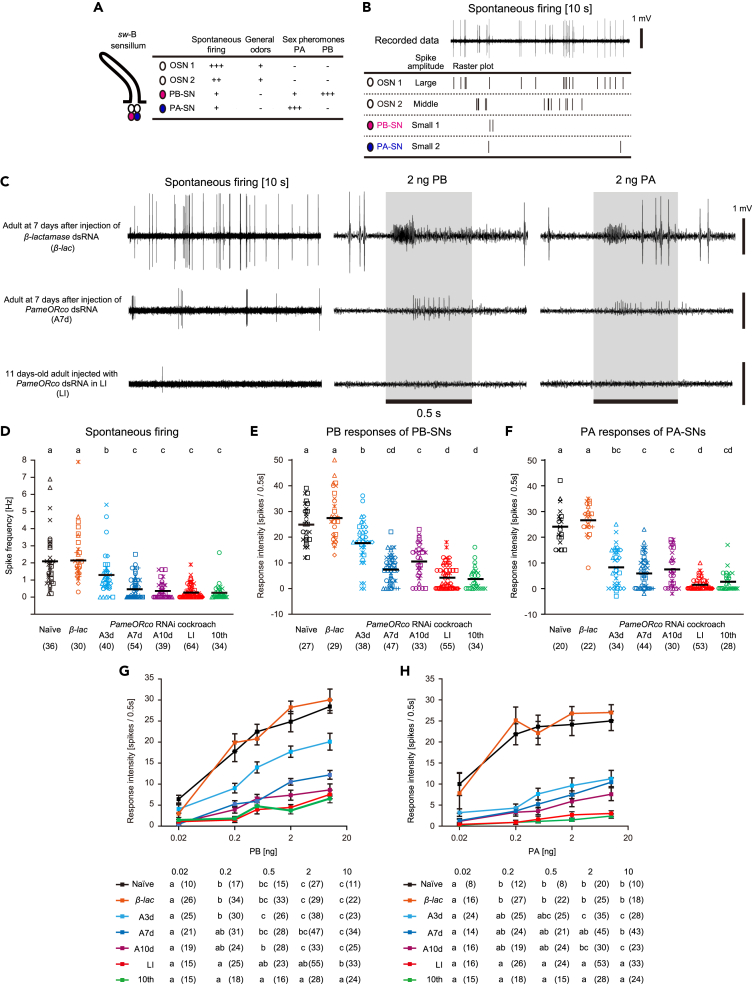
Effects of *PameORco* RNAi on the reception of sex pheromones in *sw-*B sensilla (A) Response properties of four OSNs in *sw-*B sensilla based on Boeckh and Ernst (1987). (B) Spontaneous activities of four OSNs in a *sw-*B sensillum of a *β-lac* dsRNA-injected cockroach. Spikes were sorted based on spike shapes (See STAR Methods and Figure S3). Two sex pheromone-responsive SNs (PA-SN and PB-SN) hardly exhibited spontaneous activities. (C) Typical responses of single *sw*-B sensilla from three different RNAi cockroaches. Electrophysiological traces show spontaneous activities of OSN1 and OSN2 (left), PB-responses of PB-SN (middle), and PA-responses of PA-SN (right) in single *sw*-B sensilla from three different RNAi cockroaches. (D–F) Effects of *PameORco* RNAi on activities of *sw*-B sensilla. Spontaneous spike frequencies (D), responses of PB-SNs to 2 ng of PB (E), and responses of PA-SNs to 2 ng of PA (F) in each of the RNAi conditions are summarized. Spontaneous activities and responses to each of the two sex pheromones are compared across different RNAi conditions; different letters above each column indicate significant differences (ANOVA post-hoc Tukey-Kramer test; p < 0.05). Black bars indicate means. Throughout figures, symbols of the same color and shape represent recordings from the same individuals. The numbers of recorded sensilla are noted in parentheses. (G and H) Dose-response curves of PB-SNs (G) and PA-SNs (H). The averaged response intensities of PB-SNs (G) and PA-SNs (H) to a given concentration of PB and PA are plotted with standard error (vertical bars), respectively. In each RNAi condition, response intensities to different concentrations of pheromones are statistically compared (lower tables). In the tables, the numbers of recorded sensilla are noted in parentheses, and different letters in each RNAi condition indicate significant differences (ANOVA post-hoc Tukey-Kramer test; p < 0.05).

Because all OSNs in sex pheromone-responsive *sw*-B sensilla expressed *Pame*ORco, we investigated the role of *Pame*ORco in the perception of sex pheromones in the American cockroach. At arbitrary selected days after dsRNA injection, we performed single sensillum recordings (SSRs) from single *sw*-B sensilla and recorded responses to periplanone-A (PA) and -B (PB), which are two components of *P. americana* sex pheromone (Figure 4). A *sw*-B sensillum has one PA-SN and one PB-SN in addition to two OSNs (OSN1 and OSN2), which respond to various general odors (Figure 4A; Sass, 1978; Boeckh and Ernst, 1987; Fujimura et al., 1991). In general, OSN1 and OSN2 spontaneously fired at ∼1 Hz, which corresponded to the largest and second-largest spikes, respectively, whereas PA-SN and PB-SN hardly showed spontaneous activity (Figure 4B). In *PameORco* RNAi cockroaches, the spontaneous firing of OSN1 and OSN2 significantly impaired (Figures 4C and 4D). This finding suggests that *Pame*ORco contributes to generating spontaneous activity of OSNs in the cockroach.

Because of the difficulty of discriminating small-amplitude spikes from PA-SN and PB-SN according to their spike shapes (Figures S3A and S3B), PA-evoked responses of PA-SNs and PB-evoked responses of PB-SNs were characterized by performing SSRs with a cross-adaptation odor stimuli system (Figures S3C and S3D). In brief, PA-SN responded exclusively to PA, whereas the PB-SN responded to both PB and PA (Figure 4A referred to from Boeckh and Ernst, 1987). Therefore, we recorded the PA response of PA-SN after eliminating the PA response of PB-SN by repeating stimulations with PB (Figure S3D). Our SSRs revealed that the PA responses of PA-SNs and the PB responses of PB-SNs were impaired in all *PameORco* RNAi cockroaches (Figures 4C, 4E, and 4F). The *PameORco* expression was significantly suppressed from 3 days after *PameORco* dsRNA injection (Figure 3C), but responses to PA and PB at the time point was still stronger than those at >7 days after injection. Adult cockroaches injected with *PameORco* dsRNA at the nymphal stage responded to neither PA nor PB. The effects of RNAi were maintained at least 88 days after *PameORco* dsRNA injection into the 10th instar nymph.

Next, we characterized the sensitivities of PA-SNs and PB-SNs in *PameORco* RNAi cockroaches (Figures 4G and 4H). In the control cockroaches (Naive or *β-lac* in Figure 3A), responses of PB-SNs and PA-SNs plateaued at 0.5 ng of PB and 0.2 ng of PA, respectively (Figures 4G and 4H). At 3 days after dsRNA injection, PB-SNs exhibited sufficiently strong responses to high concentrations of PB, but the sensitivity to PB was lower than that in control cockroaches. Adult cockroaches injected with *PameORco* dsRNA at nymphal stages did not respond to high concentrations of sex pheromones (LI or 10th in Figures 4G and 4H). Our results revealed that sex pheromones in the cockroach are received by functional ORs, and sensitivities and response intensities to sex pheromones depended on the post-dsRNA injection periods. Especially, the effects of *PameORco* RNAi were long-lasting and most prominent in adult cockroaches injected with *Pame*ORco dsRNA at nymphal stages.

### Silencing of the *Pame*ORco expression impaired general odor reception in a sensillum-specific manner

Next, we investigated the role of *Pame*ORco in the perception of general odors. We focused on OSNs in the *sw*-A, *sw*-C2, and *dw*-A2 sensilla whose ligands have been identified (Fujimura et al., 1991). Because the spikes were not easily distinguishable in many recorded olfactory sensilla, we characterized recorded sensilla in terms of the total number of spikes per sensillum. Based on the results of the effects of *Pame*ORco RNAi on sex pheromone reception, we compared sensillar responses to general odors among the three groups; Naive and *β-lac* dsRNA-injected cockroaches (Control), adult cockroaches at >7days after *PameORco* dsRNA injection (Adult injection), and adult cockroaches injected with *PameORco* dsRNA at the last instar stage (LI injection).

**Figure 5:**
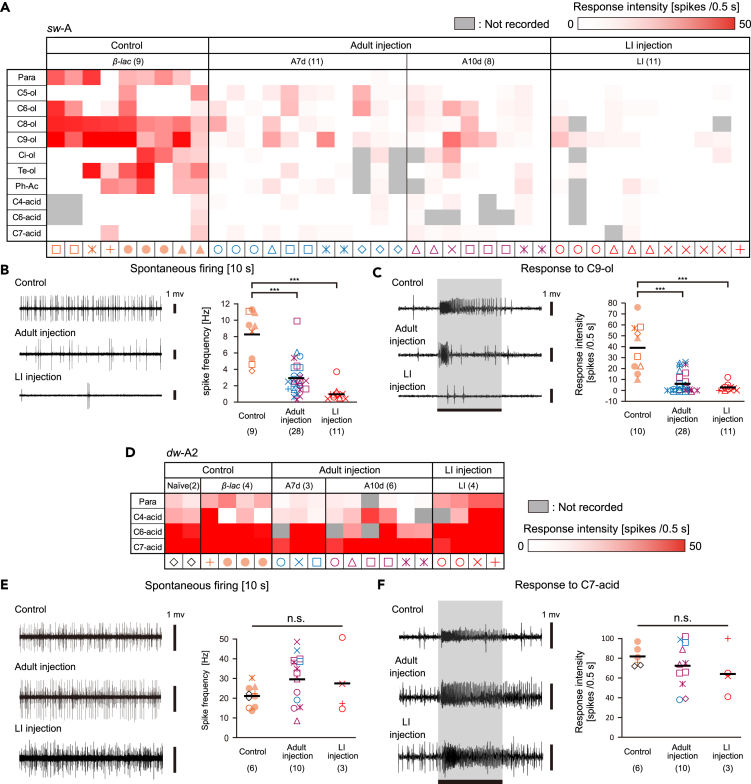
*PameORco* RNAi impairs general odor receptions in *sw*-A sensilla but not in *dw-*A2 sensilla (A) Response intensities of single *sw*-A sensilla to ten tested odorants in both Control and *PameORco* RNAi cockroaches. Responses to a given odorant are color-coded according to the response intensity. Olfactory responses of 9 sensilla from 6 Control cockroaches and of 30 sensilla from 14 *PameORco* RNAi cockroaches are summarized. (B and C) Effects of *PameORco* RNAi on activities of *sw*-A sensilla. Both spontaneous activities (B) and responses to the best ligand (C: nonanol; C9-ol) of *sw*-A sensilla were impaired in *PameORco* RNAi cockroaches. (D) Response intensities of single *dw*-A2 sensilla to the three selected odors in both Control and *PameORco* RNAi cockroaches. Among ten tested odors, OSNs in the *dw*-A2 sensilla are selectively activated by three acids (See also Figure S4A). Responses to a given odorant are color-coded according to the response intensity. Olfactory responses of 6 sensilla from 5 Control cockroaches and of 13 sensilla from 10 *PameORco* RNAi cockroaches are summarized. (E and F) Effects of *PameORco* RNAi on activities of *dw*-A2 sensilla. Both spontaneous activities (E) and responses to the best ligand (F: heptanoic acid; C7-acid) of *dw*-A2 sensilla were not affected by *PameORco* RNAi. Black bars in each plot indicate means. Throughout figures, symbols of the same color and shape correspond to recordings from the same individual. Significant differences are denoted above each plot (ANOVA post-hoc Tukey-Kramer test; ∗∗∗ = p < 0.001, n.s. = p > 0.05).

Single *sw-*A sensilla had two *Pame*ORco-positive OSNs (Figures 2A and 2E). Consistent with the previous studies, OSNs in *sw*-A sensilla in the Control group were broadly tuned to alcohols and terpenes (Figure 5A; Sass, 1983; Fujimura et al., 1991; Tateishi et al., 2020). In the Control group, the response spectra to tested odors varied among different *sw*-A sensilla, suggesting that various ORxs are expressed in OSNs (Figure 5A). In both the Adult and LI injection groups, both the spontaneous activities and olfactory responses of OSNs in tested *sw*-A sensilla were significantly decreased (Figures 5A–5C). Consistent with the results of the *sw*-B sensilla, activities of OSNs were hardly observed in the LI injection group. These results indicated that OSNs in *sw*-A sensilla perceived general odors via functional ORs.

In grooved basiconic sensilla, two subtypes, *dw-*A1 and *dw-*A2 sensilla, housed three and four ORco-negative SNs, respectively (Figrues 2D and 2E). Single *dw-*A2 sensilla were equipped with one thermosensory neuron and three OSNs, some of which tuned to hexanoic acid (C6-acid) and/or heptanoic acid (C7-acid) as observed in Control cockroaches (Figures 5D and S4A, Altner et al., 1977; Fujimura et al., 1991; Nishikawa et al., 1992). Both the spontaneous activities and responses of OSNs to acids in *dw*-A2 sensilla were maintained in *PameORco* RNAi cockroaches (Adult and LI injection groups in Figures 5D–5F).

**Figure 6:**
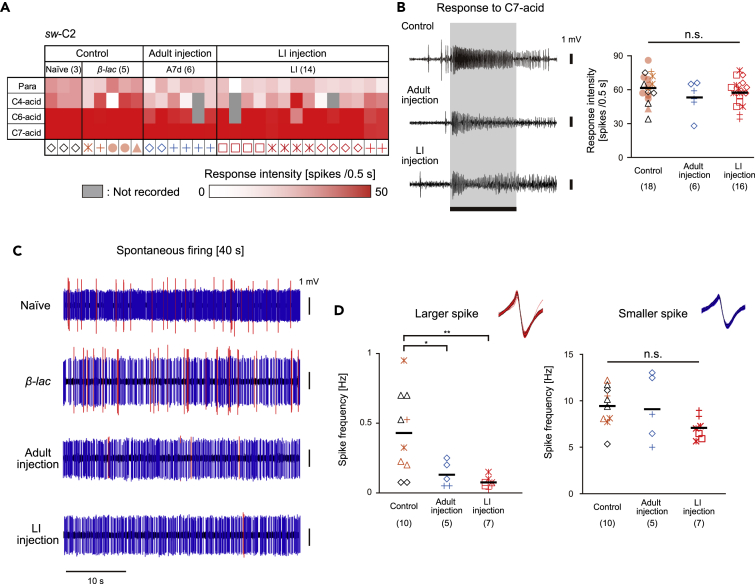
Heteromeric combination of a *Pame*ORco-positive OSN and a *Pame*ORco-negative OSN in single *sw-*C2 sensilla (A) Response intensities of single *sw*-C2 sensilla to the three selected odors in both Control and *PameORco* RNAi cockroaches. Among ten tested odors, OSNs in the *sw*-C2 sensilla were selectively activated by three acids (See also Figure S4B). Responses to a given odorant were color-coded according to the response intensity. Olfactory responses of 8 sensilla from 6 Control cockroaches and of 20 sensilla from 6 *PameORco* RNAi cockroaches are summarized. (B) Effects of *PameORco* RNAi on the acid-responses of *sw*-C2 sensilla. Responses to the best ligand (heptanoic acid; C7-acid) of *sw*-C2 sensilla were not affected by *PameORco* RNAi. (C) Typical spontaneous activities of single *sw*-C2 sensilla in both Control and *PameORco* RNAi cockroaches. In Control cockroaches, single *sw*-C2 sensilla exhibited large (red) and small (blue) amplitude spontaneous spikes. (D) Effect of *PameORco* RNAi on the spontaneous spike activities of single *sw*-C2 sensilla. *PameORco* RNAi selectively impaired the spontaneous activities of large amplitude spikes but not those of small amplitude spikes. Black bars in each plot indicate means. Throughout figures, symbols of the same color and shape represent recordings from the same individual. Significant differences are denoted above each plot (ANOVA post-hoc Tukey-Kramer test; ∗∗ = p < 0.01, ∗ = p < 0.05, n.s. = p > 0.05).

In single trichoid sensilla, a *Pame*ORco-positive OSN was consistently paired with a *Pame*ORco-negative OSN regardless of subtype; *sw-*C1 and shorter *sw-*C2 sensilla (Figures 2C and 2E). SSRs from single *sw-*C2 sensilla revealed that there were OSNs with large and small spontaneous spikes, and OSN with small spikes consistently exhibited phasic-tonic excitatory responses to C6-acid and/or C7-acid (Figures 6A–6C, S4B). Both the spontaneous activity and responses of the OSNs with small spikes to all tested acids were not affected by *PameORco* RNAi (Figures 6A–6D; one-way ANOVA test; C4-acid: p = 0.25; C6-acid: p = 0.21; C7-acid: p = 0.32), suggesting that the *Pame*ORco-negative OSN generates small spikes. Because effective ligands for the OSN with large spikes are unknown (Figures S4B), we could not precisely evaluate the effects of *PameORco* RNAi on the OSN. However, the spontaneous firing rate of the large spike significantly decreased in *PameORco* RNAi cockroaches compared with the Control group (Figures 6C and 6D). These findings indicate that a *Pame*ORco-positive OSN was consistently paired with an acid-responsive *Pame*ORco-negative OSN in single *sw-*C2 sensilla.

### Effects of PameORco RNAi on sex pheromone processing in the central brain

**Figure 7:**
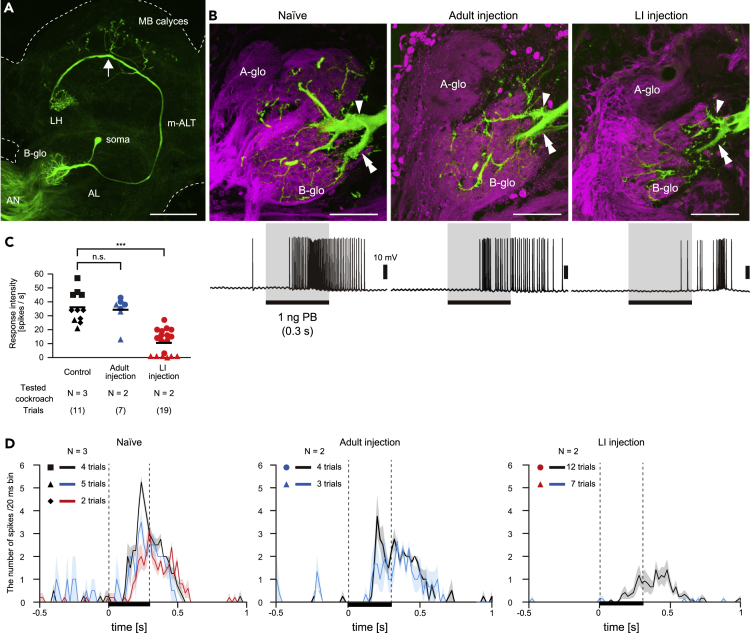
Effects of *PameORco* RNAi on sex pheromones processing in the antennal lobe (A) PB-responsive L1-PN. Uniglomerular projection neurons (PNs) with dendrites throughout the B-glomerulus (B-glo) were intracellularly stained (Green) after recordings of olfactory responses. Arrow shows the insertion site of the glass electrode. AN; antennal nerve, AL; antennal lobe, m-ALT; medial AL tract, MB; mushroom body, LH; lateral horn. (B) Arborization patterns of the recorded L1-PNs (upper panel) and their typical responses to PB (lower panels). To visualize the B-glomerulus, antennal afferents were anterogradely stained (magenta) after staining the recorded L1-PN (green). Although the responses to 1 ng of PB in Naive and *PameORco* RNAi cockroaches differed, arborization patterns of L1-PN dendrites (primary dendrite, arrowheads; major collateral, double arrowheads in upper panel) and volumes of B-glomeruli (see Figure S5) were conserved. (C) PB-responses of L1-PNs in both Naive and *PameORco* RNAi cockroaches. In cockroaches injected with *PameORco* dsRNA at the last instar stage (LI injection), the PB-responses of L1-PNs were significantly impaired. Black bars indicate means. Because PB stimulus was presented to antennae 5–8 times in each of recordings, symbols of the same color and shape represent stimulus trials from the same L1-PN. Significant differences are denoted above the plot (ANOVA post-hoc Tukey-Kramer test; ∗∗∗ = p < 0.001, n.s. = p > 0.05). (D) Temporal activity patterns of recorded L1-PNs in both Naive and *PameORco* RNAi cockroaches. The temporal activity pattern of each recorded L1-PN form a single individual is displayed as peri-stimulus time histogram with 20 ms bins. Solid lines and shaded area represent averaged responses and +/− SEM of repeated trials, respectively. Behavioral responses to PB in Naive and *PameORco* RNAi (Adult injection) cockroaches are shown in Figure S6, and Videos S5 and S6. And Scale bar = 200 μm in (A); 50 μm in (B).

To evaluate the effect of *PameORco* RNAi on brain neurons, we focused on the PB-responsive PNs, which receive sensory inputs from a large number of PB-SNs in the B-glomerulus. Among 12–15 PB-responsive PNs in the B-glomerulus, L1-PN is morphologically homologous to other uniglomerular PNs that process ordinary odorants (Nishino et al., 2018). It sends axon terminals to specific regions of the mushroom body calyx and lateral horn via the medial antennal lobe tract (mALT) (Figure 7A). We intracellularly recorded and stained L1-PNs and compared PB responses and dendritic arborizations in the B-glomerulus between Naive and *PameORco* RNAi cockroaches.

Silencing of the PB responses of PB-SNs neither affected the volume of the SN terminals (Figure S5) nor dendritic arborizations of the L1-PN dendrites in *PameORco* RNAi cockroaches (Figure 7B). It suggests silencing the *PameORco* expression did not affect the development of both peripheral sensory neurons and central brain neurons. Although PB responses of PB-SNs were significantly impaired in Adult injection group (Figure 4), L1-PNs exhibited moderately strong responses to PB, and response intensities and latencies of L1-PNs did not differ from those of Naive cockroaches (Figure 7B–7D). Thus, our results suggest that brain neurons can compensate for the impaired olfactory responses of OSNs to some extent, and it is corresponding to the result of behavioral experiment using the Adult injection group cockroaches; they displayed in an interest in PB, but the ratio of individuals which exhibited the sexual behavior (wing-raising) is significantly lower than that of Naive cockroaches (Figure S6).

In LI injection group, L1-PNs exhibited weak responses to PB and later response latencies (Figures 7B–7D). The *PameORco* dsRNA injected at the LI stage effectively impairs olfactory responses in both peripheral and central neurons in the cockroach.

## Discussion

In this study, we functionally analyzed the olfactory co-receptor in the cosmopolitan pest *P. americana* (*PameORco*) and revealed fundamental mechanisms underlying the olfactory receptions in the insect. Here, whole sequence of *PameORco* and its sensillar type-specific expression patterns were characterized. Silencing of *PameORco* via systemic RNAi impaired receptions of sex pheromones and a large variety of general odors, especially alcohols and terpenes, through OSNs in the antennal perforated basiconic and trichoid sensilla. In addition, *Pame*ORco was essential for evoking the spontaneous activities of OSNs as has been reported in other insect species (Larsson et al., 2004; Stengl and Funk, 2013; Li et al., 2016; Butterwick et al., 2018). These results suggest that *Pame*ORco not only forms a functional heteromeric complex with the ligand-specific ORx but also forms a homotetramer on its own that controls the spontaneous activities of OSNs (Nakagawa et al., 2012; Butterwick et al., 2018; Wicher and Miazzi, 2021). In this study, we also revealed RNAi can effectively suppress the *PameORco* expression during long-lasting period beyond several molts. Our study is the first to characterize the fundamental molecular process of odor transduction in the cockroach, and provide new insights into further understanding of the molecular mechanisms of peripheral and central olfactory processing in the cockroach.

We characterized *ORco* from the genome of *P. americana*. Among species in the order Blattodea, whole alignment of *ORco* has been conducted with sequences from the German cockroach *B. germanica* and two species of termites *Reticulitermes chinensis* and *Odontotermes formosanus* (Robertson et al., 2018; Gao et al., 2020; He et al., 2020). Consistent with the result of a recent nuclear phylogenomic study of the order Blattodea (Evangelista et al., 2019), *PameORco* exhibits higher homology with *ORco* of termites than that of *B. germanica* (Figure S1; Robertson et al., 2018). Because termites might have acquired eusociality by developing the chemical and pheromonal communication, analysis of *P. americana* olfactory receptors could provide new insights into the evolutionary transition from cockroaches to eusocial termites (Harrison et al., 2018). *PameORco* exhibits high homology with *ORco* in hemimetabolous insects but the low homology with *ORco* in holometabolous insects, such as *ORco* in *D. melanogaster* (*DmelORco*). However, the C-terminal region of *Pame*ORco is conserved across insect ORco, which suggests that this region might mediate the functional interactions of ORx and ORco proteins (Figure 1B, Carraher et al., 2015). In addition, the putative phosphorylation and calmodulin-binding-sites-related channel activities that have been identified in *Dmel*ORco are conserved in *Pame*ORco (Kumar et al., 2013; Carraher et al., 2015; Guo et al., 2017; Jain et al., 2021). Thus, the odor transduction mechanisms for *Dmel*ORco may be similar to those for *Pame*ORco.

We established effective experimental condition for *PameORco* RNAi in the American cockroach. *PameORco* expression was almost completely suppressed at 3 days after *PameORco* dsRNA injection, but *Pame*ORco-expressing OSNs exhibited sufficient responses to ligand odors at that time point. The reduction of OSN responses plateaued at >7 days after *PameORco* dsRNA injection (Figure 4), suggesting that the turnover rate of OR expression in OSNs is approximately one week in the cockroach. The effect of *PameORco* RNAi was the most prominent in cockroaches injected with dsRNA at nymphal stage. The strong suppression of *PameORco* expression was maintained for at least 88 days, and we did not observe recovery from the effects of RNAi in all tested cockroaches. Long-lasting and irreversible RNAi effects have also been observed when RNAi was applied to silencing genes related to visual and mechanosensory reception in the cockroach (French et al., 2015; Hennenfent et al., 2020). In other hemimetabolous insects, such as the cricket *Gryllus bimaculatus* and the firebrat *Thermobia domestica*, long-term effects of RNAi on circadian rhythm-related genes have also been reported (Uryu et al., 2013). These results suggest that RNAi may have long-lasting effects on hemimetabolous insects regardless of the gene targeted, and the RNAi technique utilize in any experiments.

The long-lasting and irreversible *PameORco* RNAi effects are very surprising because it has been believed that RNAi-mediated breakdown of target mRNA is transient, and the knockdown effect disappears after the depletion of short interfering RNA. In several insect species, mutagenesis of *ORco* by CRISPER/Cas9 induced the loss or smaller volumes of targeted glomeruli of OSNs expressing ORs (Trible et al., 2017; Fandino et al., 2019). However, our morphological analysis of a target glomerulus of PB-SNs indicated that *ORco* RNAi did not affect developmental process of OSNs (Figure S5). Therefore, we hypothesize that the possible mechanism of the long-lasting effect is piwi-mediated transcriptional silencing (Onishi et al., 2021). Piwi is a paralog of Argonaute proteins, RNA-binding proteins required for RNAi machinery, and predominantly expressed in germline cells to suppress transposon activity. Interestingly, we found that the *piwi* homolog of *P. americana* is expressed in the antennae by conducting a blast search on the transcriptome database of the cockroach antennae (GenBank ID: SRR3089536 and SRR3089537; Chen et al., 2016). Further studies are needed to reveal the molecular/cellular mechanisms underlying dsRNA-mediated long-lasting gene knockdown in long-living hemimetabolous insects.

Both anti-*Pame*ORco antiserum staining and SSRs combined with *PameORco* RNAi unambiguously revealed that the cockroaches detect the sex pheromones, PA and PB, via OSNs expressing ORx/ORco complex in *sw*-B sensilla (Figures 2 and 4). Transcriptome analysis has revealed that the two *OR* genes, *PameOR1* and *PameOR2*, are predominantly expressed in adult male antennae and are thought to be ligand-specific ORxs of sex pheromone receptors (Chen et al., 2016). To determine the sex pheromone receptors in the cockroach, functional analysis of *PameOR1* and *PameOR2* using the methods established in this study is needed. Additional studies must reveal the sex pheromone receptors in the cockroach.

Sex pheromones in the cockroach elicit the sexual behavior in adult males but not nymphs (Tobin et al., 1981). However, nymphs also have both peripheral and central neurons to process sex pheromones (Schaller-Selzer, 1984; Nishino et al., 2009; Tateishi et al., 2020). In nymphal cockroaches, PB-SNs are housed in *sw*-A sensilla (Tateishi et al., 2020), and at final molt, the sex pheromone-responsive *sw*-A sensilla elongate and transform into *sw-*B sensilla (Schaller, 1978; Watanabe et al., 2018a). The results of a previous study and our study indicate that the sensitivities of PB-SNs to PB dramatically increased according to the sensillar elongation (Figure 4G, Tateishi et al., 2020). The reported dose-response curve of PB-SNs in nymphal cockroaches is consistent with that in adult cockroaches at 3 days after *PameORco* dsRNA injection, whose ORs might be partially metabolized (Figures 3 and 4). Because the dendrites of PB-SNs expand in according to the sensillar elongation (Schaller, 1978), a greater number of receptors may be expressed on the dendrites at final molt. Thus, postembryonic changes of sex pheromone receptor expression may be linked with the sensitivities of sex pheromone-responsive OSNs, and trigger sexual behaviors in adult cockroaches.

Intracellular recordings from brain neurons using *PameORco* RNAi cockroaches revealed relationships between the activities of pre- and post-synaptic olfactory neurons. In adult cockroaches at 7 days after injection of *PameORco* dsRNA, PB-SNs exhibited impaired responses to PB, whereas post-synaptic L1-PNs exhibited sufficient responses to 1 ng of PB, which were comparable to those in Naive cockroaches, and exhibited impaired behavioral responses to the PB. Responses of PB-SNs to 1 ng of PB in the cockroaches at 7 days after injection of *PameORco* dsRNA were equivalent to the responses to 20 pg of PB in Naive cockroaches (Figure 4G), suggesting that a low concentration of sex pheromone effectively activates brain neurons in Naive cockroach. In fact, 0.1 pg of PB can elicit sufficient excitatory responses in L1-PN of Naive cockroaches and elicited sexual behaviors (Boeckh and Selsam, 1984; Domae et al., 2019). The high sensitivity of the L1-PN is partly explained by the high sensory convergence of PB-SNs; approximately 37,000 PB-SNs send axons into the B-glomerulus and synapse onto a L1-PN (Boeckh and Ernst, 1987). Nevertheless, L1-PNs hardly responded to PB in the cockroach injected with *PameORco* dsRNA at the LI stage, suggesting that *PameORco* RNAi performed in the nymphal stage resulted in the near-complete suppression of sex pheromone processing.

The single-walled trichoid sensilla are common in many insect species, but their functions vary; these sensilla are specifically tuned sex pheromones in many moth species (Ng et al., 2020) and broadly tuned to a large variety of odors in *D. melanogaster* (Munch and Galizia, 2016) and the locust *Locusta migratoria* (Li et al., 2016; Yang et al., 2012; You et al., 2016). Regardless of the functional differences among insect species, OSNs in the trichoid sensilla only express ORs, with the exception of the cockroach as shown in this study. In the cockroach, trichoid sensilla are classified into *sw*-C1 and *sw*-C2 sensilla based on differences in their lengths and target glomeruli (Watanabe et al., 2012). In single *sw*-C2 sensilla, a *Pame*ORco-positive OSN always pairs with a *Pame*ORco-negative OSN that is tuned to acids (Figures 2 and 6). Acids are specific ligands for IRs (Ai et al., 2010; Raji et al., 2019), suggesting that an OR-OSN and an IR-OSN are co-housed in a sensillum. Except for a subtype of *double-walled* coeloconic sensilla (ac4B sensilla) in *D. melanogaster*, single sensilla with OSNs expressing ORs and IRs have not been reported in other insects to date (Benton et al., 2009; Munch and Galizia, 2016). To confirm the specific features of the sensilla, the distributions and functions of IRs in the cockroach should be analyzed in the future. Although we were not able to record from *sw*-C1 sensilla in this study, single *sw*-C1 sensilla are equipped with two unique OSNs termed ON-cell and OFF-cell, which are excited by the onset and offset of a given odor stimulus, respectively (Burgstaller and Tichy, 2011; Tichy and Hellwig, 2018). Our anti-*Pame*ORco antiserum staining suggests that ON-OFF information of a given odor stimulus may be received by different types of olfactory receptors.

*Pame*ORco-negative and acid-tuned OSNs in grooved basiconic sensilla strongly suggest that they also express IRs. In the cockroach brain, general olfactory signals detected by perforated basiconic sensilla are independently processed from those processed by trichoid and grooved basiconic sensilla through the two parallel olfactory pathways; OSNs in two different classes of sensilla project to different groups of glomeruli in the antennal lobe (AD glomeruli and PV glomeruli in Figure S5A) and synapse onto two different classes of PNs, which terminate in different areas of the mushroom body, and each area has dendrites of different classes of Kenyon cells (Watanabe et al., 2010a, 2012, 2017; Takahashi et al., 2019). Therefore, with the exception of *Pame*ORco-positive OSNs in trichoid sensilla, the OR- and IR-related olfactory circuits in the cockroach brain are ambiguously segregated. In the moth (Fandino et al., 2019) and fruit fly (Grabe and Sachse, 2018), odor information received by ORs and IRs elicits different odor-guided behaviors, suggesting that OR- and IR-related circuits have different functions. The simple but sophisticated olfactory processing from peripheral to higher brain centers in the cockroach is one of the best systems for studying how animals encode various odors and translate them into odor-guided behaviors via OR- and IR-related parallel circuits.

### Limitations of the study

Our study revealed the distribution of ORco and its function in odor reception in the antennae of the American cockroach, *P. americana*. *ORco*-impaired cockroaches exhibited partial anosmia, especially to sex pheromones, in a sensillum-specific manner. Electrophysiological experiment and RT-qPCR suggest systemic RNAi of *PameORco* chronically and irreversibly suppressed odor reception via odorant receptors (ORs). Because we can design dsRNA to target species-specific gene, RNAi is considered a robust approach for assessing the physiological actions of repellent or attractants in IPM (Gondhalekar et al., 2021). In this study, adult cockroaches at >7 days after *PameORco* dsRNA injection displayed in an interest in the sex pheromones, but the ratio of individuals which exhibited sexual behaviors is significantly lower than that of Naive cockroaches. It is highly corresponding to the results of phylogenetically related German cockroach; behavioral responses to the sex pheromone and food odors were partially disrupted in adult cockroaches at 3 days after *ORco* dsRNA injection (He et al., 2020). Our results in the American cockroach suggest that the injection of a small amount of *PameORco* dsRNA to nymphal cockroaches more effectively impairs odor and sex pheromone receptions in adults, and the effect is long-lasting and irreversible. It needs the further behavioral studies using the adult cockroaches which received dsRNA injection at nymphal stage. In addition, it needed to determine the most efficient way to deliver dsRNA to nymphal cockroaches (e.g., baits including the dsRNA) under field conditions.

## STAR★Methods

### Key resource table

**Table:** 

REAGENT or RESOURCE	SOURCE	IDENTIFIER
**Antibodies**		

Guinea pig anti-*Pame*ORco antiserum	This paper	N/A
Rabbit polyclonal anti-peroxidase (anti-HRP)	Sigma-Aldrich	Cat#P7899; RRID: AB_261181
Goat anti-Rabbit IgG (H + L) Secondary Antibody, Alexa Fluor™ Plus 488	Thermo Fisher Scientific	Cat#A32731; RRID: AB_2633280
Goat anti-Guinea Pig IgG (H + L) Secondary Antibody, Alexa Fluor™ 555	Thermo Fisher Scientific	Cat#A21435; RRID: AB_2535856

**Chemicals**, peptides, and recombinant proteins		

n-hexane (Hex)	FUJIFILM Wako	Cat# 082-06,901
periplanone-A (PA)	(Kuwahara and Mori, 1990a)	N/A
periplanone-B (PB)	(Kuwahara and Mori, 1990b)	N/A
n-paraffin-oil (Para)	FUJIFILM Wako	Cat#126-05535
n-pentanol (C5-ol)	FUJIFILM Wako	Cat# 019-03,653
n-hexanol (C6-ol)	FUJIFILM Wako	Cat# 087-00,513
n-octanol (C8-ol)	FUJIFILM Wako	Cat# 152-00,133
n-nonanol (C9-ol)	TCI	Cat# N0292
Cineol (Ci-ol)	FUJIFILM Wako	Cat# 051-03,972
α-terpineol (Te-ol)	TCI	Cat# T0984
phenyl acetate (Ph-Ac)	TCI	Cat# A0043
butyric acid (C4-acid)	FUJIFILM Wako	Cat# 029-05,393
hexanoic acid (C6-acid)	TCI	Cat# H0105
heptanoic acid (C7-acid)	FUJIFILM Wako	Cat# 084-00,182

**Deposited data**		

*PameORco* isoform 1	This paper	GenBank ID: LC657818
*PameORco* isoform 2	This paper	GenBank ID: LC657819
*PameEf1alpha*	This paper	GenBank ID: LC657820

**Oligonucleotides**		

Primer for full-length cDNA amplification for *PameORco* isoform 1, forward: 5′-CTTGCAGATGTACAAGGCACG-3′	This paper	N/A
Primer for full-length cDNA amplification for *PameORco* isoform 1, reverse: 5′-GTAGAATGTGATGTTGTAGGTCTAGTTG-3′	This paper	N/A
Primer for full-length cDNA amplification for *PameORco* isoform 2, forward: 5′-CTTGCAGATGTACAAGGCACG-3′	This paper	N/A
Primer for full-length cDNA amplification for *PameORco* isoform 2, reverse: 5′-CAGGCATGCTTGTATGTTTATCAC-3′	This paper	N/A
Primer with T7 promoter sequence (underlined) used to amplify the template DNAs for *in vitro* transcription for *PameORco,* forward: 5′-CTAATACGACTCACTATAGGGAGCAACA TCGGAGACACTTATG-3′	This paper	N/A
Primer with T7 promoter sequence (underlined) used to amplify the template DNAs for *in vitro* transcription for *PameORco,* reverse: 5′-CTAATACGACTCACTATAGGGCGAAACAAAGTG ACTTTCAGGTAC-3′	This paper	N/A
Primer with T7 promoter sequence (underlined) used to amplify the template DNAs for *in vitro* transcription for *E.coli β-lac,* forward: 5′-GAATTAATACGACTCACTATAGGGTGAGTATT CAACATTTCCGTGTC-3′	This paper	N/A
Primer with T7 promoter sequence (underlined) used to amplify the template DNAs for *in vitro* transcription for *E.coli β-lac,* reverse: 5′-GAATTAATACGACTCACTATAGGGCAG CACTGCATAATTCTCTTACTG-3′	This paper	N/A
Primer used for RT-qPCR for *PameORco*, forward: 5′-CGTTGATTCGGATAATGCAGATG-3′	This paper	N/A
Primer used for RT-qPCR for *PameORco,* reverse: 5′-GTTCAGAAAGAGCAGCAGGAAC-3′	This paper	N/A
Primer used for RT-qPCR for *PameEf1alpha,* forward: 5′-CGACTTTATTGCTCAGGTTATTGTG-3′	This paper	N/A
Primer used for RT-qPCR for *PameEf1alpha,* reverse: 5′-GATTTTCCTCAGTAGTCTTACCAGTAC-3′	This paper	N/A

**Software and algorithms**		

Amira 6.0	Thermo Fisher Scientific	3D Visualization & Analysis Software | Thermo Fisher Scientific - JP
Spike 2	Cambridge Electronic Design Limited	http://ced.co.uk/products/spkovin
Bellcurve for Microsoft Excel	Social Survey Research Information	https://bellcurve.jp/ex/
MAFFT	(Katoh and Standley, 2013)	http://www.geneious.com/
BoxShade	Bioinformatics Group BBSRC Institute	https://embnet.vital-it.ch/software/BOX_form.html
MEGA X	(Kumar et al., 2016)	https://github.com/rambaut/figtree/releases

### Resource availability

#### Lead contact

Further information and requests for resources and reagents should be directed to and will be fulfilled by the lead contact, Hidehiro Watanabe, Ph. D (nabehide@fukuoka-u.ac.jp).

#### Material availability

Antiserum against to *Pame*ORco generated in this study is available from the lead contact upon request.

### Experimental model and subject details

#### Animals

Tenth instar, last instar (LI: 11th instar), and adult male cockroaches *P. americana* with intact antennae were obtained from laboratory colonies maintained at 27 ± 1 °C under a 12:12 light-dark cycle at Fukuoka University. The nymphal stages were identified according to the lengths of the body and hind tibia (Gier, 1947). For experiments, cockroaches were collected from colonies immediately after molt and individually reared in separate glass tubes.

### Method details

#### Molecular cloning of *PameORco*

We cloned an *ORco* homolog expressed in the cockroach. First, we conducted a tblastn search in the genomic DNA sequence database of *P*. *americana* (GenBank ID: PGRX00000000.1) using the deduced amino acid sequence of *Drosophila melanogaster* ORco protein (GenBank ID: Q9VNB5). Next, we performed RT-PCR to amplify cDNA fragments containing the full-length open reading frame (ORF) of the gene using primers designed at the predicted 5′ and -3′′-untranslated regions of *PameORco*. Complementary DNA (cDNA) was prepared according to Watanabe et al. (2010b). Nucleotide sequences of the primers are listed in in key resource table. The nucleotide sequences of the obtained cDNAs of *PameORco* isoform 1 and 2 were registered in GenBank (GenBank ID: LC657818 and LC657819).

#### Structural and phylogenetic analyses of insect ORco homologs

The deduced amino acid sequence of *PameORco* isoform 2 was aligned with those of ORco homologs of other insect species using the MAFFT algorithm (Katoh and Standley, 2013) and refined by manual inspection using the Geneious Prime program (v2021.1.1) created by Biomatters (available from http://www.geneious.com/). The alignment was visualized using the BoxShade program (v3.2.1; available from https://embnet.vital-it.ch/software/BOX_form.html). A phylogram was constructed using the bootstrapped maximum likelihood method (1000 bootstrap replicates) in MEGA X (v10.2.6; Kumar et al., 2016), and visualized using the FigTreev1.4.4 (available from https://github.com/rambaut/figtree/releases).

#### Immunohistochemistry

The polyclonal anti-*Pame*ORco antiserum was generated in a guinea pig against the 14-a.a. synthetic peptide (NH_2_-C+VSANTNNPAATGDD-COOH) located within the second intracellular loop of the protein (Figure 1; residues 263 to 276) (Eurofins genomics, Tokyo, Japan). We also used a polyclonal antibody generated in rabbit against peroxidase from horseradish (anti-HRP; Sigma-Aldrich Cat# P7899, RRID: AB_261181). We previously showed that the anti-HRP antibody selectively labeled sensory neurons in cockroach antennae (Watanabe et al., 2018a).

Immunohistochemistry protocols followed those used for the cockroach antennae with modifications (Watanabe et al., 2018a). The two antennae of a day-old adult cockroaches were removed, and each antenna was divided into several fragments. Each fragment was manually sliced along the longitudinal axis with a razor blade. Sliced fragments were fixed in 4% paraformaldehyde with 0.1 M phosphate buffer (PB, pH 7.4) at 4 °C for a day. After washing in PB saline (PBS: 0.14 M NaCl in 0.01 M PB, pH 7.4) with 1% Triton X-100 (PBST), the fragments were blocked for an hour using 5% normal goat serum (NGS; 143-06561, Fujifilm Wako, Osaka, Japan) in PBST with 0.25% bovine serum albumin (PBST-BSA; A2153, Sigma-Aldrich, St. Louis, MO). Antennal fragments were then incubated with a mixture solution of polyclonal anti-HRP antibody (rabbit) and anti-*Pame*ORco antiserum (guinea pig) at 4 °C for 4 days. The anti-HRP antibody and anti-*Pame*ORco antiserum were diluted 1:5000 and 1:1000 in PBST-BSA with 2% NGS, respectively. On the following day, the fragments were washed again in PBST-BSA and then incubated with the mixture solution of Alexa488-labeled secondary anti-rabbit IgG antibody (diluted 1:1000; A32731, Thermo Fisher Scientific; RRID: AB_2633280) and Alexa 555-labeled secondary anti-guinea pig IgG antibody (diluted 1:1000; A21435, Thermo Fisher Scientific; RRID: AB_2535856) in PBST-BSA with 2% NGS at 4 °C for 4 days. The fragments were then finally washed in PBST-BSA, dehydrated with an ascending ethanol series (from 50% to 100%), and cleared in methyl salicylate. No signal was detected when specimens were labeled with only secondary antibodies (data not shown).

#### Preparation and injection of double-stranded RNA (dsRNA)

DsRNA for *PameORco* and *Escherichia coli β-lactamase* (*β-lac*) genes were synthesized by T7 RNA polymerase-based *in vitro* transcription. A partial cDNA fragment of each target gene with T7 promoter sequences at both ends was used as a template for *in vitro* transcription. The details for each template are provided below.

##### PameORco

A 424-bp partial cDNA fragment of *PameORco* encoding the C-terminal region (including the sixth and seventh transmembrane helixes) of the protein was used as a template for *in vitro* transcription. The nucleotide sequence of *PameORco* dsRNA is specific for *P. americana*, and the *PameORco* dsRNA targets both mRNA isoforms. The cDNA was amplified by PCR from a plasmid containing a partial cDNA clone of *PameORco*. PCR was carried out with a pair of gene-specific primers (GSPs) with the T7 promoter sequence at the 5′ end (Key resource table).

##### *E. coli* β-lac

A 369-bp partial cDNA fragment of *E. coli β-lac* was used as a template for *in vitro* transcription. The cDNA fragment was amplified by PCR from the pGEM-T easy vector (Promega, Madison, WI, USA) with a pair of GSPs with the T7 promoter sequence at the 5′ end (Key resource table).

All PCRs were carried out using the Q5 High-Fidelity DNA polymerase (New England Biolabs, Tokyo, Japan), and the PCR products were purified using the QIAquick PCR Purification Kit (Qiagen, Tokyo, Japan). Nucleotide sequences of the primers were listed in key resource table. *In vitro* transcription was carried out using the HiScribe T7 High Yield RNA Synthesis Kit (New England Biolabs, Tokyo, Japan) per the manufacturer’s instructions. Approximately 1 μg of template DNA was added to a 20 μL reaction, and incubated at 37°C for ∼16 hr. The dsRNAs were purified by lithium chloride precipitation. The synthesized dsRNAs were diluted into the nuclease-free phosphate-buffered saline to adjusted the dsRNA concentration to 0.8 μg/μL and were stored at −30°C.

For systemic RNAi of *PameORco*, we prepared 4 days-old adult males or 7 days-old nymphs (Figure 3A). After anesthetizing the cockroaches on ice, 5 μL of dsRNA solution (4 μg of dsRNA in total) was manually injected into the dorsal region of the head capsule using a 27 G needle attached to a 10-μL glass syringe. The cockroaches injected with dsRNA were separately reared in isolated glass tubes, and provided food and water *ad libitum*. Adult cockroaches were used for experiments at 3, 7 or 10 days after dsRNA injection. The nymphal cockroaches injected with dsRNA were reared until they became adults and used at 11 days after their final molt (Figure 3A). In control experiments, 11 days-old virgin males were used as Naive cockroaches.

#### Quantitative reverse transcription PCR (RT-qPCR)

The RT-qPCR analysis was conducted following the methods of Watanabe et al. (2018b) with modifications. A pair of antennae was homogenized in 500 μL of the TRIzol Reagent (Life Technologies, CA, USA) using a beads homogenizer (μT-01, TAITEC, Saitama, Japan) at 4600 r/min for 90 s at room temperature. The total RNA was extracted from each sample according to the manufacturer’s instructions. After RNA extraction, genomic DNA was digested with Recombinant DNase I (5 U/reaction; TaKaRa, Shiga, Japan). After heat-inactivation of the enzyme (75°C, 10 min), each RNA sample was reverse transcribed using the High-Capacity cDNA Reverse Transcription Kit (Life Technologies) according to the manufacturer’s instruction. A 1.5 μL of total RNA solution was added to the 10 μL reaction.

The relative quantification of target genes was carried out using the KAPA SYBR Fast qPCR Kit (Kapa Biosystems, MA, USA) and the Thermal Cycler Dice Real Time System (TaKaRa, Shiga, Japan). Gene expression levels were measured using the standard curve method. The expression levels of *PameORco* mRNA were normalized to that of *PameEf1α* (GenBank ID: LC657820) using the 2^−ΔΔCt^ method. The nucleotide sequences of the primers are listed in key resource table.

#### Single sensillum recording from OSNs in single olfactory sensilla

An ice-anesthetized cockroach was immobilized ventral-side-up on an acrylic plate. To prevent movement, the body and legs were gently mounted using low-melting wax, the neck was immobilized with small acrylic plates, and the antennae were fixed using the wax. The antennae were observed through the light microscope at 500× magnification (AZ100, Nikon, Tokyo, Japan).

We performed single sensillum recordings (SSRs) from arbitrarily selected olfactory sensilla on the ventral surface of the flagellum. Sensillar types were identified under the light microscope based on the shape and hair length. For recording, a tungsten indifferent electrode was manually inserted into the head capsule near the ipsilateral compound eye. A borosilicate glass microelectrode pulled by a laser puller (P-2000; Sutter Instruments, Novato, CA, USA) was filled with the cockroach saline (210.2 mM NaCl, 3.1 mM KCl, 1.8 mM CaCl_2_, 0.2 mM NaH_2_PO_4_, 1.8mM Na_2_HPO_4_, pH 7.2). The tip of the glass electrode was inserted into the basal cavity of the sensillum using a micromanipulator. After observation of the spontaneous activities of OSNs, olfactory stimuli were presented to the antenna. Electrical signals were processed by a preamplifier (MEZ-8201; Nihon Kohden, Tokyo, Japan) and a main AC/DC amplifier (EX-1; Dagan Corporation, Minneapolis, MN, USA), and displayed on an oscilloscope. The AC signals were filtered (bandpass 100 Hz to 1 kHz) and recorded with a Power Lab data acquisition system at a sampling rate of 10 kHz (Power Lab 8/35; AD Instruments Japan Inc., Nagoya, Japan). In each specimen, we repeatedly recorded olfactory responses from different sensilla.

After recordings, two antennae were removed and homogenized in the TRIzol Reagent for RNA extraction. After removing two antennae, anterograde staining of antennal afferents was conducted (Watanabe et al., 2010a, 2018a). The head capsule was detached from the body and fixed on a dish. The proximal cut ends of antennal nerve were inserted into a tapered glass electrode containing 10% aqueous solution of micro-ruby (D7162, Thermo Fisher Scientific). Contact between the specimen and the dye was maintained in a humid chamber at 4 C˚ overnight. The stained brain was then dissected from the head capsule. The isolated brain was fixed, dehydrated, and cleared.

#### Olfactory stimulation

We used 10 general odors for the classification of OSN types (Key resouce table; Fujimura et al., 1991; Tateishi et al., 2020) and two purified synthesized sex pheromones (PA and PB; Kuwahara and Mori, 1990a, 1990b). Both PA and PB were diluted in hexane at a concentration of 0.1 ng/μL as stock solutions. Each sex pheromone solution was placed on an aluminum plate (15 × 5 mm) and was dried for more than 1 min to evaporate the solvent. Therefore, their concentrations were denoted as dry weights. General odors were diluted in paraffin oil at a concentration of 10 mM, and 20 μL of the odorant solution was added to a piece of filter paper (15 × 5 mm). Immediately before recordings, the aluminum plates and the filter papers loaded with odorants were separately inserted into glass pipettes.

Fresh air was cleaned and dried with charcoal and silica-gel filters. The air stream was maintained at 1 L/min using a flowmeter. The main tube was tandemly connected to two electric-driven three-way solenoid valves (Figure S3C; Valve 1 and 2) which were independently operated by Powerlab. During the inter-stimulus period, the constant air which passed through the two valves and the blank glass pipette flowed over the antenna (top panel of Figure S3C). During the general odor stimulation period, the constant air stream was stopped and the air stream from the other outlet of Valve 1 passed through the glass pipette containing a given odorant. The tip of the glass pipette was positioned approximately 1 cm apart from the recording sensillum. The general odor stimulus was presented for 0.5-s period and each odorant was presented two to five times with > 40-s intervals. After stimulation, a new glass pipette containing another odorant was attached to the outlet of Valve 1.

To discriminate the PA response of PA-SN and the PB response of PB-SN, cross-adaptation experiments were performed. A glass pipette containing PB and one containing PA were connected to the outlets of Valve 1 and Valve 2, respectively. In the experiment, PA was presented at a concentration equal to PB. The tips of two glass pipettes were positioned approximately 1 cm apart from the recording sensillum. During the sex pheromone stimuli period, the recording sensillum received three successive PB stimuli and then one PA stimulus with inter-stimulus intervals of 0.5 s (Figure S3D). Each sex pheromone stimulus was presented for 0.5-s periods. The cross-adaptation experiments were performed with > 2-min intervals. PA-SN and PB-SN easily adapt to PA and PB, respectively. Although low to high concentrations of sex pheromones were presented, each concentration was presented to the recording sensillum only once during each recording period. The air around the preparation was continuously exhausted through a duct behind the recording electrode. We regarded the timing of the solenoid valve gating as the onset of odor stimulation.

#### Data analysis of SSRs

In each SSR, different shapes of spikes from different OSNs were concurrently recorded. Although the spike amplitudes were distinguishable in several traces, the spikes were not easily distinguishable in many SSRs. We grouped spikes with different shapes in each SSR and calculated the increase in total spike frequency from the spontaneous frequency as follows; R−R0, where R0 and R were the total numbers of spikes during the 0.5-s period before and after the onset of odor stimulation, respectively. The average response intensity was defined as the response intensity to a given odorant. Spontaneous spikes were counted during the 10-s period (in perforated basiconic and grooved basiconic sensilla) or 40-s period (in trichoid sensilla) before the onset of all given odor stimuli, and the spontaneous firing rate was determined by converting the averaged numbers to Hz.

Each of spikes derived from OSNs in single *sw-*B sensilla and *sw*-C2 sensilla was sorted using the spike sorting function of Spike 2 ver. 8.08 (CED, Cambridge, UK) (Figures 4, 6, and S3). We extracted each of spikes with the duration of 2 msec-period based on its spike peak. And then, principal component analysis was performed in all recorded spikes according to the spike shapes. Each spike was plotted in a three-dimensional space using the first three principal components (PC1 - PC3), and clustered (Figure S3B). Because spikes from PB-SN and PA-SN was difficult to segregate by their shapes, spikes pre-adapted to PB stimulus and those post-adapted to PA stimulus were regarded as PB-elicited spikes of PB-SN and PA-elicited spikes of PA-SN, respectively.

In electrophysiological experiments, statistical differences were determined by one-way ANOVAs and post-hoc Tukey-Kramer test using BellCurve for Excel (Social Survey Research Information, Tokyo, Japan).

#### Intracellular recording and staining of L1-PN

A glass microelectrode was filled with 8% Lucifer Yellow (Sigma-Aldrich) in 1 M LiCl_2_. The input resistances were set 30-50 MΩ. A cockroach, briefly anesthetized by carbon dioxide, was fixed onto a handmade acrylic chamber with wax. The head was positioned in a shallow chamber and fixed with low melting point wax. A small rectangular window was cut in the head cuticle between the compound eyes, muscles and trachea were removed to expose the brain surface. The brain was immersed in cockroach saline to form an electrical bridge with an indifferent silver rod (diameter, 300 μm) that was inserted into a cavity between the circumesophageal connectives. For intracellular recording and subsequent staining, the electrode was inserted into the axon just below the point where two calyces meet (Figure 7A). Electrical signals were amplified by a DC amplifier (MEX-8301, Nihon Kohden, Japan), displayed in an oscilloscope, and then digitized. L1-PNs were readily identifiable based on their spontaneous activities and responsiveness to PB. The 1 ng of PB stimulus was presented to antennae for 0.3-s periods 5-8 times with intervals of >30 s. Statistical differences were determined by one-way ANOVAs and post-hoc Tukey-Kramer test.

After recording olfactory responses, the L1-PN was filled with Lucifer Yellow by injecting a hyperpolarizing current. After staining, the electrode was removed from the brain, and the head capsule was fixed on a wax plate. Anterograde staining of antennal afferents was then conducted as described the above section. The double-stained brain was fixed, dehydrated, and cleared.

#### LSM observations and three-dimensional reconstruction of the B-glomerulus

The cleared specimens were examined with a confocal laser scanning microscope (LSM-510; Carl Zeiss, Jena, Germany) equipped with Argon and Helium-Neon lasers. Neurons labeled by Alexa488 or Lucifer Yellow were visualized using an Argon laser with a band-pass emission filter (505–530 nm), whereas those labeled Alexa555 or micro-ruby were visualized using a Helium-Neon laser with a long-pass emission filter (>560 nm). Antennal sensilla were visualized via the differential interference contrast (DIC) imaging function of the LSM. Images were obtained using three different objectives: a Plan-Apochromat 20×/0.8 objectives for low magnification images, and an oil-immersion Plan-Neofluar 40×/1.3 and Plan-Apochromat 63×/1.4 objectives were used for high magnification images. Optical sections were captured at intervals of 0.7–1.2 μm thickness with 1024 × 1024 pixels. The contrast and brightness of all images were adjusted using Adobe Photoshop CS3 and Illustrator CS3. The orientations and positions of the brain are shown relative to the body axis.

For the three-dimensional reconstruction of the B-glomerulus, a series of TIFF-formatted optical sections of macroglomeruli were processed using image processing software (Figure S5B; Amira 6.0, Thermo Fisher Scientific). The boundary of B-glomerulus was manually traced with the aid of the anterogradely stained OSN afferents. The volume of the 3-D reconstructed B-glomerulus was calculated by the function attached in Amira 6.0. Volumes of B-glomeruli in three RNAi groups were statistically compared by Kruskal-wallis test.

#### Behavioral experiment

We compared sexual behaviors to PB between Naive and *PameORco* RNAi cockroaches. We collected 4 days-old adult virgin males and divided them into Naive and *PameORco* RNAi (Adult injection) groups. In the Naive group, two or three cockroaches were reared together in a plastic cup (φ = 8 cm (bottom), height = 8 cm) spread a filter paper, and provided a paper shelter, food and water for 7 days. In the Adult injection group, two or three cockroaches injected 4 μg of *PameORco* dsRNA were reared together in a plastic cup for 7days. After then, we removed the paper shelter, food and water from the plastic cup, and performed behavioral experiment at three hours later. We observed cockroach behaviors to PB using an infrared camera (FDR-AX60, Sony, Tokyo, Japan) under the dark situation. At 4 min after the onset of recording, 1 ng of PB on a filter paper (15 × 5 mm) was gently placed on the plastic cup, and recorded behaviors to PB during 4-min period (Videos S5 and S6). After recording, tested cockroaches were again placed a new plastic cup, and were received the same behavioral experiment at 7 days later.

We evaluated the behavior of individual cockroach during the 2-min period after presentation of PB. We identified levels of sexual behavior of *P. americana* according to the previous studies as follows: 0: no response, 1: waving of antennae, 2: waving of antennae and oriented locomotion, 3: waving of antennae, oriented locomotion, partial wing-raising and wing-fluttering response, 4: waving of antennae, oriented locomotion, wing-raising, abdominal extension (Figure 6S; Silverman 1977; Yang et al., 1998). We statistically compared the ratio of cockroaches which exhibit wing-raising behavior (levels 3 and 4) between Naive and *PameORco* RNAi groups using the Fisher’s exact test.

### Quantification and statistical analysis

To validate the effectiveness of *PameORco* RNAi, we statistically compared experimental data obtained from individuals at different timepoints after dsRNA injection. We used the one-way ANOVAs and post-hoc Tukey-Kramer test to assess the results of RT-qPCR and electrophysiological experiments, the Kruskal-wallis test for morphological analysis of B-glomerulus, and the Fisher’s exact test for behavioral experiments. Statistical significance was calculated using BellCurve for Microsoft Excel (Social Survey Research Information, Tokyo, Japan). Black bars within the scatterplots indicate means. Error bars in Figures 4G and 4H, and shaded areas in Figure 7D represent as mean ± SEM The asterisks indicate significant differences between groups (n.s > P = 0.05, ∗ = P < 0.05, ∗∗ = P < 0.01, ∗∗∗ = P <0.001). The different letters above each of plots indicate significant differences according to multiple comparisons with Tukey-Kramer test (P < 0.05).

## Data Availability

Section 1: All raw data reported in this paper will be shared by the lead contact upon request. Section 2: This paper does not report original code. Section 3: Any additional information required to reanalyze the data reported in this paper is available from the lead contact upon request.

## Supplemental information

Document S1. Figures S1–S6

Video S1. PameORco distribution pattern, related to Figure 2A

Video S2. PameORco distribution pattern, related to Figure 2B

Video S3. PameORco distribution pattern, related to Figure 2C

Video S4. PameORco distribution pattern, related to Figure 2D

Video S5. Behavioral responses to 1 ng PB in Naïve cockroaches, related to Figures 7 and S6. The video plays at double speed

Video S6. Behavioral responses to 1 ng PB in PameORco RNAi cockroaches, related to Figures 7 and S6 The video plays at double speed

## References

[bib1] Ai M., Min S., Grosjean Y., Leblanc C., Bell R., Benton R., Suh G.S.B. (2010). Acid sensing by the *Drosophila* olfactory system. Nature.

[bib2] Altner H., Sass H., Altner I. (1977). Relationship between structure and function of antennal chemo-hygro-and thermoreceptive sensilla in *Periplaneta americana*. Cell Tissue Res..

[bib3] Batra S., Corcoran J., Zhang D.D., Pal P., K P.U., Kulkarni R., Lofstedt C., Sowdhamini R., Olsson S.B. (2019). A functional agonist of insect olfactory receptors: behavior, physiology and structure. Front. Cell. Neurosci..

[bib4] Benton R., Sachse S., Michnick S.W., Vosshall L.B. (2006). Atypical membrane topology and heteromeric function of *Drosophila* odorant receptors in vivo. PLoS Biol..

[bib5] Benton R., Vannice K.S., Gomez-Diaz C., Vosshall L.B. (2009). Variant ionotropic glutamate receptors as chemosensory receptors in *Drosophila*. Cell.

[bib6] Boeckh J., Ernst K.D. (1987). Contribution of single unit analysis in insects to an understanding of olfactory function. J. Comp. Physiol. A..

[bib7] Boeckh J., Selsam P. (1984). Quantitative investigation of the odour specificity of central olfactory neurones in the American cockroach. Chem. Senses.

[bib8] Burgstaller M., Tichy H. (2011). Functional asymmetries in cockroach ON and OFF olfactory receptor neurons. J. Neurophysiol..

[bib9] Butterwick J.A., Del Marmol J., Kim K.H., Kahlson M.A., Rogow J.A., Walz T., Ruta V. (2018). Cryo-EM structure of the insect olfactory receptor Orco. Nature.

[bib10] Carey A.F., Carlson J.R. (2011). Insect olfaction from model systems to disease control. Proc. Natl. Acad. Sci. U S A.

[bib11] Carraher C., Dalziel J., Jordan M.D., Christie D.L., Newcomb R.D., Kralicek A.V. (2015). Towards an understanding of the structural basis for insect olfaction by odorant receptors. Insect. Biochem. Mol. Biol..

[bib12] Chen Y., He M., Li Z.Q., Zhang Y.N., He P. (2016). Identification and tissue expression profile of genes from three chemoreceptor families in an urban pest, *Periplaneta americana*. Sci. Rep..

[bib13] DeGennaro M., McBride C.S., Seeholzer L., Nakagawa T., Dennis E.J., Goldman C., Jasinskiene N., James A.A., Vosshall L.B. (2013). Orco mutant mosquitoes lose strong preference for humans and are not repelled by volatile DEET. Nature.

[bib14] Do D.C., Zhao Y., Gao P. (2016). Cockroach allergen exposure and risk of asthma. Allergy.

[bib15] Domae M., Iwasaki M., Mizunami M., Nishino H. (2019). Functional unification of sex pheromone-receptive glomeruli in the invasive Turkestan cockroach derived from the genus *Periplaneta*. Neurosci. Lett..

[bib16] Evangelista D.A., Wipfler B., Bethoux O., Donath A., Fujita M., Kohli M.K., Legendre F., Liu S., Machida R., Misof B. (2019). An integrative phylogenomic approach illuminates the evolutionary history of cockroaches and termites (Blattodea). Proc. Biol. Sci..

[bib17] Fandino R.A., Haverkamp A., Bisch-Knaden S., Zhang J., Bucks S., Nguyen T.A.T., Schroder K., Werckenthin A., Rybak J., Stengl M. (2019). Mutagenesis of odorant coreceptor Orco fully disrupts foraging but not oviposition behaviors in the hawkmoth *Manduca sexta*. Proc. Natl. Acad. Sci. U S A.

[bib18] French A.S., Meisner S., Liu H., Weckstrom M., Torkkeli P.H. (2015). Transcriptome analysis and RNA interference of cockroach phototransduction indicate three opsins and suggest a major role for TRPL channels. Front. Physiol..

[bib19] Fujimura K., Yokohari F., Tateda H. (1991). Classification of antennal olfactory receptors of the cockroach, *Periplaneta americana* L. Zool. Sci..

[bib20] Fusca D., Kloppenburg P. (2021). Odor processing in the cockroach antennal lobe-the network components. Cell Tissue Res..

[bib21] Gao Y., Huang Q., Xu H. (2020). Silencing orco impaired the ability to perceive trail pheromones and affected locomotion behavior in two termite species. J. Econ. Entomol..

[bib22] Gier H.T. (1947). Growth rate in the cockroach *Periplaneta americana* (Linn). Ann. Entomol. Soc. Am..

[bib23] Gondhalekar A.D., Appel A.G., Thomas G.M., Romero A. (2021). A review of alternative management tactics employed for the control of various cockroach species (order: blattodea) in the USA. Insects.

[bib24] Grabe V., Sachse S. (2018). Fundamental principles of the olfactory code. Biosystems.

[bib25] Guo H., Kunwar K., Smith D. (2017). Odorant receptor sensitivity modulation in *Drosophila*. J. Neurosci..

[bib26] Harrison M.C., Jongepier E., Robertson H.M., Arning N., Bitard-Feildel T., Chao H., Childers C.P., Dinh H., Doddapaneni H., Dugan S. (2018). Hemimetabolous genomes reveal molecular basis of termite eusociality. Nat. Ecol. Evol..

[bib27] He P., Ma Y.F., Wang M.M., Wang H., Dewer Y., Abd El-Ghany N.M., Chen G.L., Yang G.Q., Zhang F., He M. (2020). Silencing the odorant coreceptor (Orco) disrupts sex pheromonal communication and feeding responses in *Blattella germanica*: toward an alternative target for controlling insect-transmitted human diseases. Pest Manag. Sci..

[bib28] Hennenfent A., Liu H., Torkkeli P.H., French A.S. (2020). RNA interference supports a role for Nanchung-Inactive in mechanotransduction by the cockroach, *Periplaneta americana*, tactile spine. Invertebr. Neurosci..

[bib29] Jain K., Lavista-Llanos S., Grabe V., Hansson B.S., Wicher D. (2021). Calmodulin regulates the olfactory performance in *Drosophila melanogaster*. Sci. Rep..

[bib30] Katoh K., Standley D.M. (2013). MAFFT multiple sequence alignment software version 7: improvements in performance and usability. Mol. Biol. Evol..

[bib31] Kumar B.N., Taylor R.W., Pask G.M., Zwiebel L.J., Newcomb R.D., Christie D.L. (2013). A conserved aspartic acid is important for agonist (VUAA1) and odorant/tuning receptor-dependent activation of the insect odorant co-receptor (Orco). PLoS One.

[bib32] Kumar S., Stecher G., Tamura K. (2016). MEGA7: molecular evolutionary genetics analysis version 7.0 for bigger datasets. Mol. Biol. Evol..

[bib33] Kuwahara S., Mori K. (1990). Synthesis of both the enantiomers of hauptmann's periplanone-A and clarification of the structure of Persoons's periplanone-A. Tetrahedron.

[bib34] Kuwahara S., Mori K. (1990). Synthesis of (-)-periplanone-B, A sex pheromone component of the American cockroach (*Periplaneta americana*). Tetrahedron.

[bib35] Larsson M.C., Domingos A.I., Jones W.D., Chiappe M.E., Amrein H., Vosshall L.B. (2004). Or83b encodes a broadly expressed odorant receptor essential for *Drosophila* olfaction. Neuron.

[bib36] Li S., Zhu S., Jia Q., Yuan D., Ren C., Li K., Liu S., Cui Y., Zhao H., Cao Y. (2018). The genomic and functional landscapes of developmental plasticity in the American cockroach. Nat. Commun..

[bib37] Li Y., Zhang J., Chen D., Yang P., Jiang F., Wang X., Kang L. (2016). CRISPR/Cas9 in locusts: successful establishment of an olfactory deficiency line by targeting the mutagenesis of an odorant receptor co-receptor (Orco). Insect Biochem. Mol. Biol..

[bib38] Lundin C., Kall L., Kreher S.A., Kapp K., Sonnhammer E.L., Carlson J.R., von Heijne G., Nilsson I. (2007). Membrane topology of the *Drosophila* OR83b odorant receptor. FEBS Lett..

[bib39] Munch D., Galizia C.G. (2016). DoOR 2.0--comprehensive mapping of *Drosophila melanogaster* odorant responses. Sci. Rep..

[bib40] Nakagawa T., Pellegrino M., Sato K., Vosshall L.B., Touhara K. (2012). Amino acid residues contributing to function of the heteromeric insect olfactory receptor complex. PLoS One.

[bib41] Ng R., Wu S.T., Su C.Y. (2020). Neuronal compartmentalization: a means to integrate sensory input at the earliest stage of information processing?. Bioessays.

[bib42] Nishikawa M., Yokohari F., Ishibashi T. (1992). Response characteristics of two types of cold receptors on the antennae of the cockroach, *Periplaneta americana* L. J. Comp. Physiol. A..

[bib43] Nishino H., Iwasaki M., Paoli M., Kamimura I., Yoritsune A., Mizunami M. (2018). Spatial receptive fields for odor localization. Curr. Biol..

[bib44] Nishino H., Yoritsune A., Mizunami M. (2009). Different growth patterns of two adjacent glomeruli responsible for sex-pheromone processing during postembryonic development of the cockroach *Periplaneta americana*. Neurosci. Lett..

[bib45] Onishi R., Yamanaka S., Siomi M.C. (2021). piRNA- and siRNA-mediated transcriptional repression in *Drosophila*, mice, and yeast: new insights and biodiversity. EMBO. Rep..

[bib46] Pai H.-H., Ko Y.C., Chen E.R. (2003). Cockroaches (*Periplaneta american*a and *Blattella germanica*) as potential mechanical disseminators of Entamoeba histolytica. Acta Trop..

[bib47] Persoons C.J., Ritter F.J., Verwiel P.E.J., Hauptmann H., Mori K. (1990). Nomenclature of American cockroach sex-pheromones. Tetrahedron Lett..

[bib48] Raji J.I., Melo N., Castillo J.S., Gonzalez S., Saldana V., Stensmyr M.C., DeGennaro M. (2019). *Aedes aegypti* mosquitoes detect acidic volatiles found in human odor using the IR8a pathway. Curr. Biol..

[bib49] Robertson H.M., Baits R.L., Walden K.K.O., Wada-Katsumata A., Schal C. (2018). Enormous expansion of the chemosensory gene repertoire in the omnivorous German cockroach *Blattella germanica*. J. Exp. Zool. Part B.

[bib50] Sass H. (1978). Olfactory receptors on the antenna of *Periplaneta*: response constellations that encode food odors. J. Comp. Physiol. A..

[bib51] Sass H. (1983). Production, release and effectiveness of two female sex pheromone components of *Periplaneta americana*. J. Comp. Physiol. A..

[bib52] Schaller-Selzer L. (1984). Physiology and morphology of the larval sexual pheromone-sensitive neurones in the olfactory lobe of the cockroach, *Periplaneta americana*. J. Insect Physiol..

[bib53] Schaller D. (1978). Antennal sensory system of *Periplaneta americana* L.: distribution and frequency of morphologic types of sensilla and their sex-specific changes during postembryonic development. Cell Tissue Res..

[bib54] Seelinger G., Gagel S. (1985). On the function of sex pheromone components in *Periplaneta americana*: improved Odour Source localization with periplanone-A. Physiol. Entmol..

[bib55] Silverman J.M. (1977). Patterns of response to sex pheromone by young and mature adult male cockroaches, *Periplaneta americana*. J. Insect Physiol..

[bib56] Stengl M., Funk N.W. (2013). The role of the coreceptor Orco in insect olfactory transduction. J. Comp. Physiol. A..

[bib57] Sun H., Liu F., Ye Z., Baker A., Zwiebel L.J. (2020). Mutagenesis of the orco odorant receptor co-receptor impairs olfactory function in the malaria vector *Anopheles coluzzii*. Insect Biochem. Mol. Biol..

[bib58] Takahashi N., Nishino H., Domae M., Mizunami M. (2019). Separate but interactive parallel olfactory processing streams governed by different types of GABAergic feedback neurons in the mushroom body of a basal insect. J. Neurosci..

[bib59] Tateishi K., Nishimura Y., Sakuma M., Yokohari F., Watanabe H. (2020). Sensory neurons that respond to sex and aggregation pheromones in the nymphal cockroach. Sci. Rep..

[bib60] Tichy H., Hellwig M. (2018). Independent processing of increments and decrements in odorant concentration by ON and OFF olfactory receptor neurons. J. Comp. Physiol. A..

[bib61] Tichy H., Zeiner R., Traunmuller P., Martzok A., Hellwig M. (2020). Developing and testing of an air dilution flow olfactometer with known rates of concentration change. J. Neurosci. Methods..

[bib62] Tobin T.R., Seelinger G., Bell W.J. (1981). Behavioral responses of male *Periplaneta americana* to periplanone B, a synthetic component of the female sex pheromone. J. Chem. Ecol..

[bib63] Trible W., Olivos-Cisneros L., McKenzie S.K., Saragosti J., Chang N.C., Matthews B.J., Oxley P.R., Kronauer D.J.C. (2017). Orco mutagenesis causes loss of antennal lobe glomeruli and impaired social behavior in ants. Cell.

[bib64] Uryu O., Kamae Y., Tomioka K., Yoshii T. (2013). Long-term effect of systemic RNA interference on circadian clock genes in hemimetabolous insects. J. Insect Physiol..

[bib65] Wada-Katsumata A., Silverman J., Schal C. (2013). Changes in taste neurons support the emergence of an adaptive behavior in cockroaches. Science.

[bib66] Watanabe H., Nishino H., Nishikawa M., Mizunami M., Yokohari F. (2010). Complete mapping of glomeruli based on sensory nerve branching pattern in the primary olfactory center of the cockroach *Periplaneta americana*. J. Comp. Neurol..

[bib67] Watanabe H., Haupt S.S., Nishino H., Nishikawa M., Yokohari F. (2012). Sensillum-specific, topographic projection patterns of olfactory receptor neurons in the antennal lobe of the cockroach *Periplaneta americana*. J. Comp. Neurol..

[bib68] Watanabe H., Nishino H., Mizunami M., Yokohari F. (2017). Two parallel olfactory pathways for processing general odors in a cockroach. Front. Neural Circuit..

[bib69] Watanabe H., Koike Y., Tateishi K., Domae M., Nishino H., Yokohari F. (2018). Two types of sensory proliferation patterns underlie the formation of spatially tuned olfactory receptive fields in the cockroach *Periplaneta americana*. J. Comp. Neurol..

[bib70] Watanabe T., Takeuchi H., Kubo T. (2010). Structural diversity and evolution of the N-terminal isoform-specific region of ecdysone receptor-A and -B1 isoforms in insects. BMC Evol. Biol..

[bib71] Watanabe T., Ugajin A., Aonuma H. (2018). Immediate-early promoter-driven transgenic reporter system for neuroethological research in a hemimetabolous insect. eNeuro.

[bib72] Wicher D., Miazzi F. (2021). Functional properties of insect olfactory receptors: ionotropic receptors and odorant receptors. Cell Tissue Res..

[bib73] Yang H.T., Chow Y.S., Peng W.K., Hsu E.L. (1998). Evidence for the site of female sex pheromone production in Periplaneta americana. J. Chem. Ecol..

[bib74] Yang Y., Krieger J., Zhang L., Breer H. (2012). The olfactory co-receptor Orco from the migratory locust (*Locusta migratoria*) and the desert locust (*Schistocerca gregaria*): identification and expression pattern. Int. J. Biol. Sci..

[bib75] You Y., Smith D.P., Lv M., Zhang L. (2016). A broadly tuned odorant receptor in neurons of trichoid sensilla in locust, *Locusta migratoria*. Insect Biochem. Mol. Biol..

