# Supplementary material for: Silencing the odorant receptor co-receptor impairs olfactory reception in a sensillum-specific manner in the cockroach
Source: iScience. 2022 Apr 20;25(5):104272. doi: 10.1016/j.isci.2022.104272 (PMC9065313; doi:10.1016/j.isci.2022.104272)
Supplement: Document S1. Figures S1–S6 [file mmc1.pdf]

**iScience, Volume *25***

## Supplemental information

# Silencing the odorant receptor co-receptor impairs olfactory reception in a sensillum-specific manner in the cockroach

**Kosuke Tateishi, Takayuki Watanabe, Hiroshi Nishino, Makoto Mizunami, and Hidehiro Watanabe**

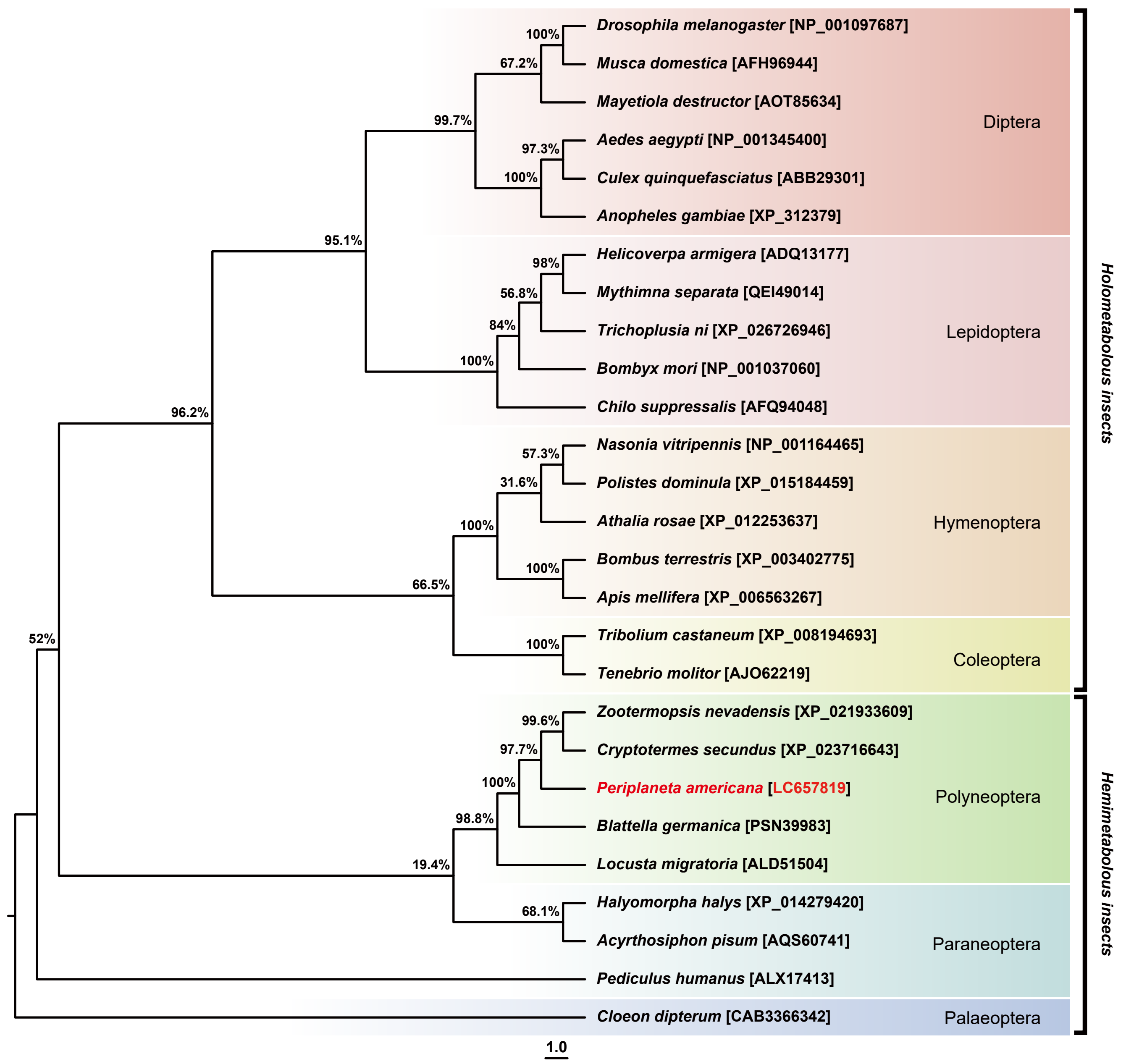


**Figure S1. Molecular phylogenetic tree of insect ORco proteins, related to Figure 1.**

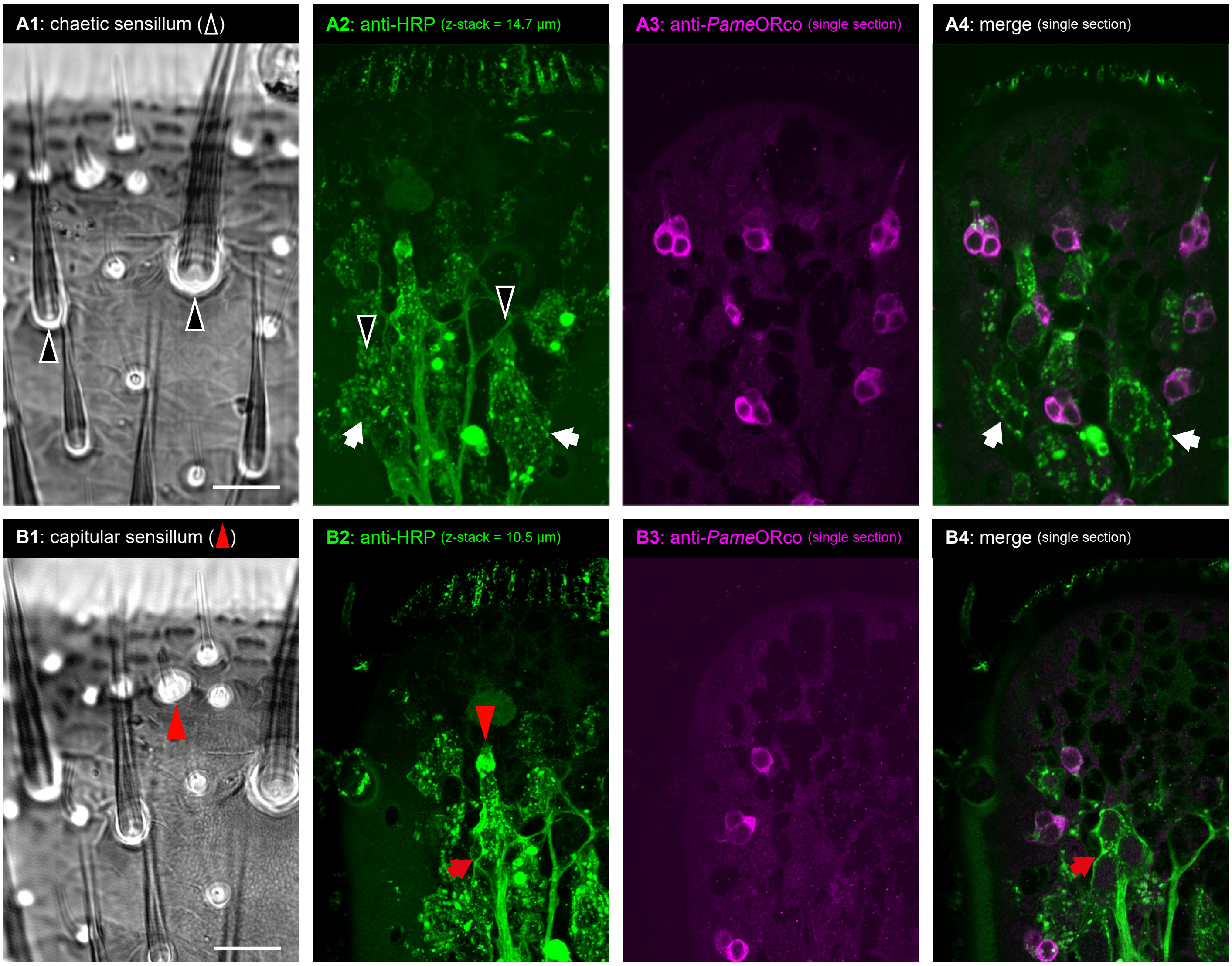


**Figure S2. Anti-*Pame*ORco antiserum did not label un-olfactory sensory neurons, related to Figure 2.**

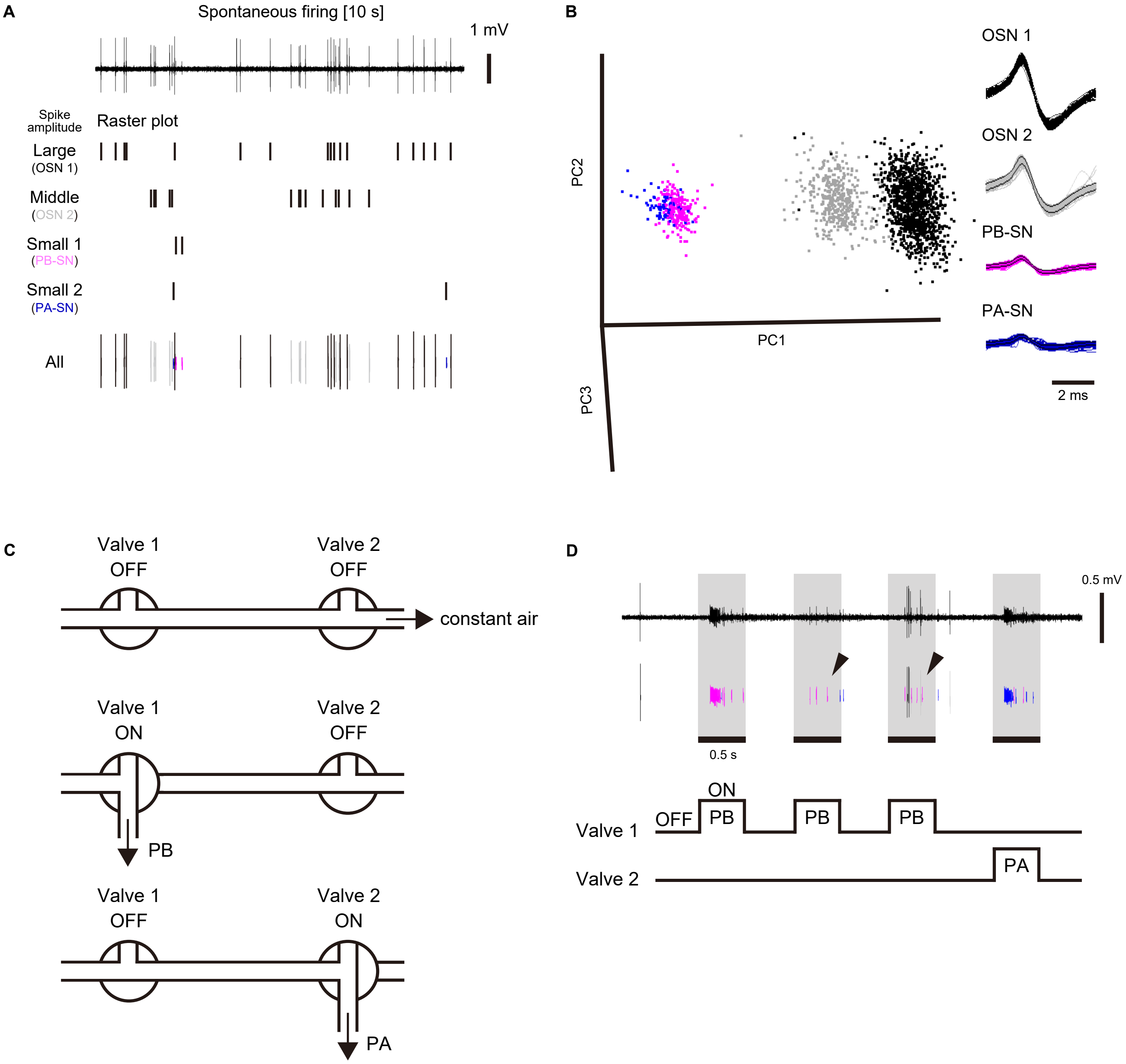


**Figure S3. Identification of spikes from four OSNs in single *sw*-B sensilla, related to Figure 4.**

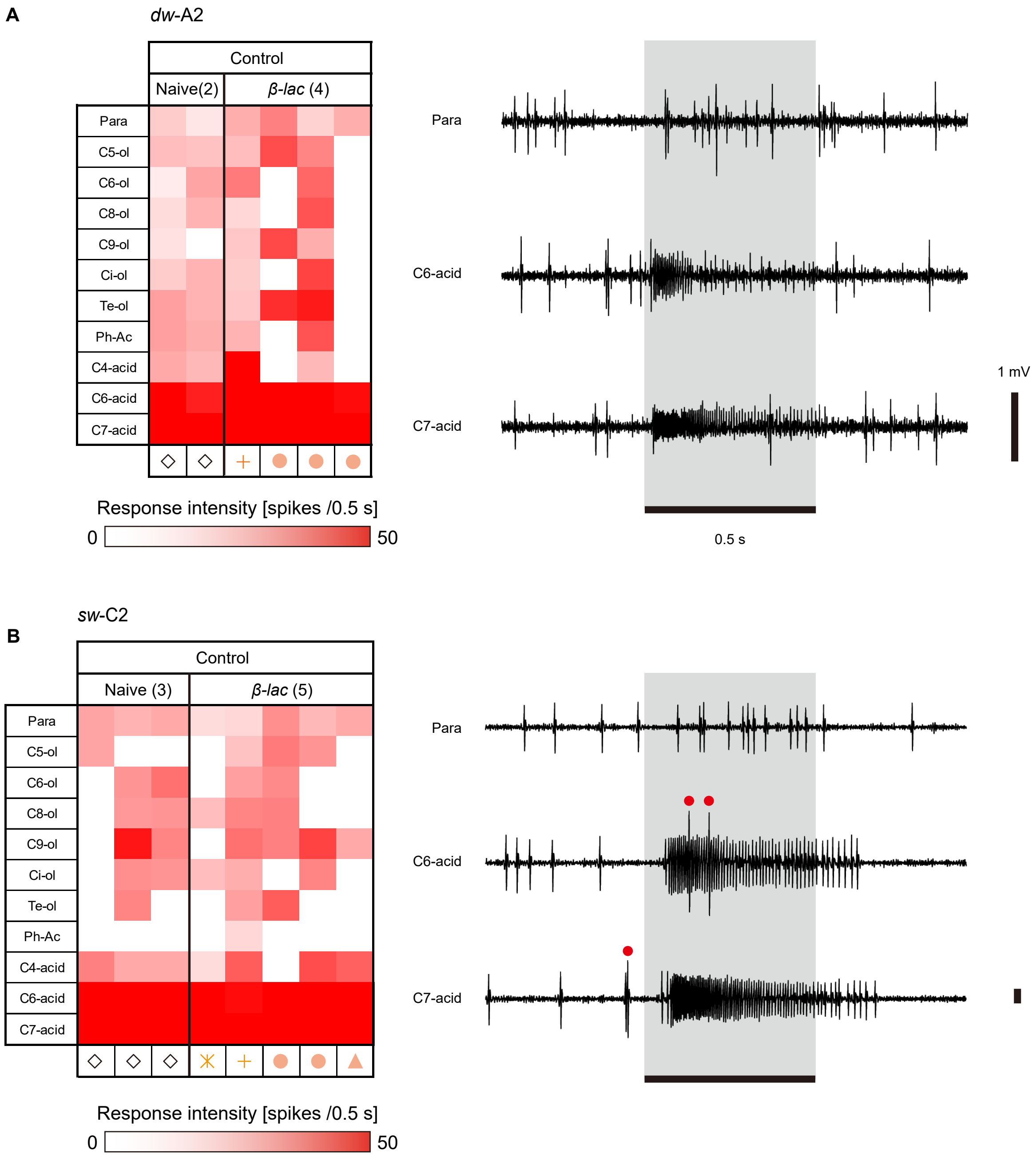


**Figure S4. Olfactory response spectra of single *dw*-A2 (A) and *sw*-C2 (B) sensilla, related to Figures 5 and 6.**

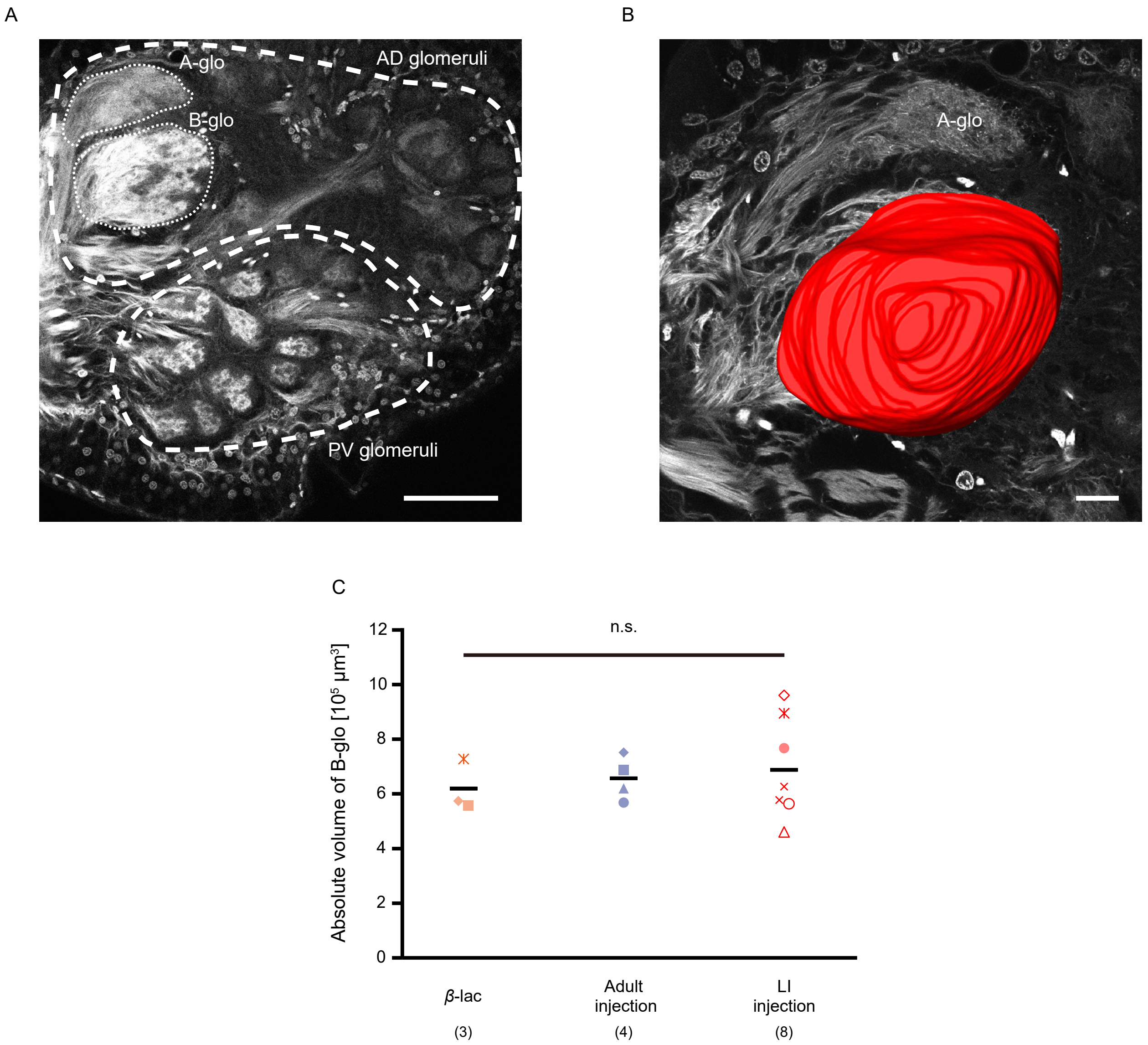


**Figure S5. Volumes of B-glomeruli in the *PameORco* RNAi cockroaches, related to Figure 7.**

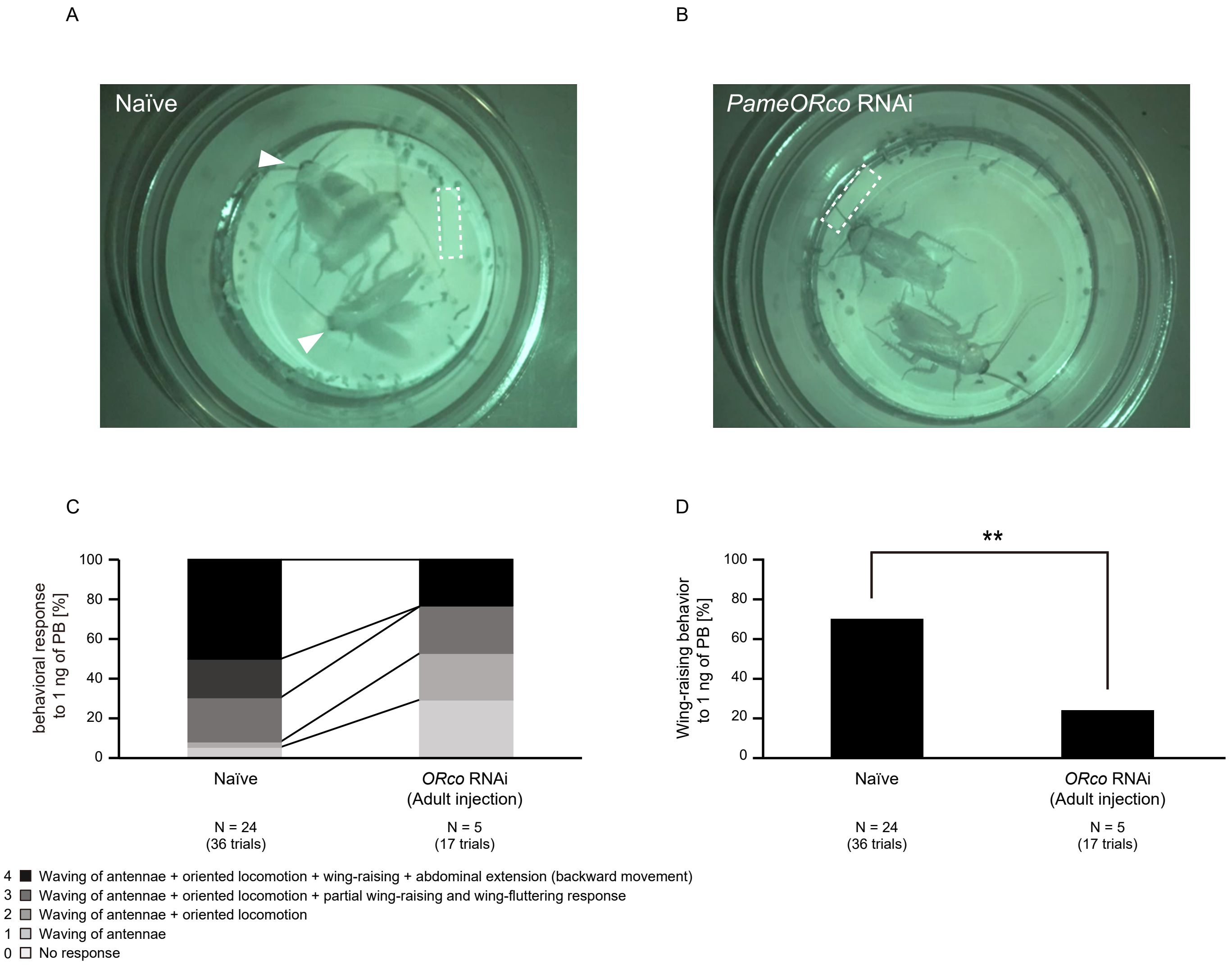


**Figure S6. Behavioral responses to PB in *PameORco* RNAi cockroaches, related to Figure 7.**
